# A colorimetric test for the evaluation of the insecticide content of LLINs used on Bioko Island, Equatorial Guinea

**DOI:** 10.1186/s12936-021-03967-w

**Published:** 2021-11-10

**Authors:** Harparkash Kaur, Elizabeth Louise Allan, Teunis A. Eggelte, Guillermo A. García, Feliciano Monti

**Affiliations:** 1 London School of Hygiene and Tropical Medicine, Keppel St, London, WC1E 7HT UK; 2 Medical Care Development International, Silver Spring, MD USA; 3 Medical Care Development International, Malabo, Equatorial Guinea; 4 Present Address: US Agency for International Development Embassy of the United States of America, Yangon, Myanmar

**Keywords:** Malaria, Vector control, Insecticide-treated nets, Insecticide concentration, Insecticidal activity, Colorimetric Test Kit

## Abstract

**Background:**

Insecticide-treated nets and indoor residual spraying of insecticides are used as the vector control interventions in the fight against malaria. Measuring the actual amount of deposits of insecticides on bed nets and walls is essential for evaluating the quality and effectiveness of the intervention. A colorimetric “Test Kit” designed for use as a screening tool, able to detect the type II pyrethroids on fabrics and sprayed walls, was used for the first time to detect deltamethrin on long-lasting insecticidal nets (LLINs) deployed on Bioko Island, Equatorial Guinea.

**Methods:**

LLINs were analysed using the colorimetric Test Kit performed in situ, which leads to the formation of an orange-red solution whose depth of colour indicates the amount of type II pyrethroid on the net. The kit results were validated by measuring the amount of extracted insecticide using high-performance liquid chromatography (HPLC) with diode array detection (DAD).

**Results:**

Deltamethrin concentration was determined for 130 LLINs by HPLC–DAD. The deltamethrin concentration of these nets exhibited a significant decrease with the age of the net from 65 mg/m^2^ (< 12 months of use) to 31 mg/m^2^ (> 48 months; p < 0.001). Overall, 18% of the nets being used in households had < 15 mg/m^2^ of deltamethrin, thus falling into the “Fail” category as assessed by the colorimetric Test Kit. This was supported by determining the bio-efficacy of the nets using the WHO recommended cone bioassays. The Test Kit was field evaluated in situ and found to be rapid, accurate, and easy to use by people without laboratory training. The Test Kit was shown to have a reliable linear relationship between the depth of colour produced and deltamethrin concentration (R^2^ = 0.9135).

**Conclusion:**

This study shows that this colorimetric test was a reliable method to assess the insecticidal content of LLINs under operational conditions. The Test Kit provides immediate results and offers a rapid, inexpensive, field-friendly alternative to the complicated and costly methods such as HPLC and WHO cone bioassays which also need specialist staff. Thus, enabling National Malaria Control Programmes to gain access to effective and affordable monitoring tools for use in situ.

**Supplementary Information:**

The online version contains supplementary material available at 10.1186/s12936-021-03967-w.

## Background

Insecticide-treated nets (ITNs) have been the most effective intervention in reducing malaria mortality and morbidity across Africa since 2000 [1, 2] and are recommended as one of the main tools for malaria control [3]. The introduction of long-lasting insecticidal nets (LLINs) has been a significant innovation [4], and LLINs are now the only type of mosquito net recommended by the World Health Organization (WHO) Global Malaria Programme [5]. LLINs are impregnated during manufacturing with an insecticide that is bound to the fibres of the net and is released slowly over time, providing long-term protection. LLINs protect people from malaria in several ways by providing a physical barrier preventing human-vector contact, killing mosquitoes that come into contact with the treated fabric, and by providing an excito-repellent effect of insecticides [6], which further reduces human-vector contact. LLIN use provides both a direct protective effect for the individuals sleeping under them and a community effect when universal coverage of at least one net for every two people is achieved [1, 7]. Currently, global coverage with LLINs is actively promoted as a safe, reliable, and effective method for malaria prevention and control by the WHO, The United Nations International Children’s Emergency Fund, The United Nations Development Programme, The World Bank, and the Roll Back Malaria Partnership.

The success of LLINs as an effective malaria intervention is dependent on their durability and use. The WHO recommends that programmes regularly monitor and evaluate the durability of LLINs based on net survivorship, fabric integrity, and assessing the efficacy of the insecticide (bio-efficacy) [8–12]. Routine monitoring of LLINs is critical to ensure programmes strategize and coordinate LLIN replacement campaigns and provide information about their effective life span under everyday usage. The efficacy of the insecticide residue is mainly determined by bioassay and chemical analysis, with chemical analysis being the only way to quantify the amount of insecticide on the net. Cone bioassays are the most commonly used method that exposes insecticide susceptible mosquito strains to impregnated netting material for determining the residual insecticidal activity of the net [13]. However, bioassay testing requires skilled staff with access to laboratory and insectary facilities. According to WHO guidelines for malaria, these susceptibility tests should also be supplemented with additional methods like genetic and biochemical testing [11]. Moreover, in situ net testing using bioassays can be challenging to perform, with nets typically removed from the homes for analysis. Chemical analysis utilizing either gas chromatography (GC) or high-performance liquid chromatography (HPLC) are both costly in terms of equipment and requires highly trained personnel with expertise using validated analysis procedures [14, 15]. As a result, many malaria-endemic countries lack the resources and infrastructure to monitor and evaluate LLIN durability.

The WHO recommends expanding test procedures for operationally meaningful data for monitoring insecticides used in vector control [11]. Developing inexpensive and straightforward colorimetric field tests is vital for effectively monitoring LLINs, particularly in field settings where nets can be tested at home. A simple, rapid, and inexpensive colorimetric field test was developed to detect three type II pyrethroids (i.e., deltamethrin, α-cypermethrin, and λ-cyhalothrin) in the field on hung LLINs while conserving their integrity. Except for λ-cyhalothrin, these compounds are frequently used in LLINs (e.g., Vestergaard's PermaNet 2.0, BASF’s interceptor, Permanet 3.0, Olyset Plus). The test is based on detecting cyanide ions released from type II pyrethroids [16]; hence it cannot detect the other extensively used type I pyrethroid, namely permethrin.

This study aimed to investigate only the insecticidal activity of LLINs distributed on Bioko Island using the cone bioassays and two different approaches, a colorimetric test, and HPLC with diode array detection (DAD), to assess the insecticide concentration. The performances of these two approaches in estimating the insecticide content were confirmed and compared against each other. In addition, the physical durability of LLINs was assessed to estimate the need and frequency of net replacement to maintain adequate coverage among the beneficiary populations.

## Methods

### Study area and sample collection

**Fig. 1:**
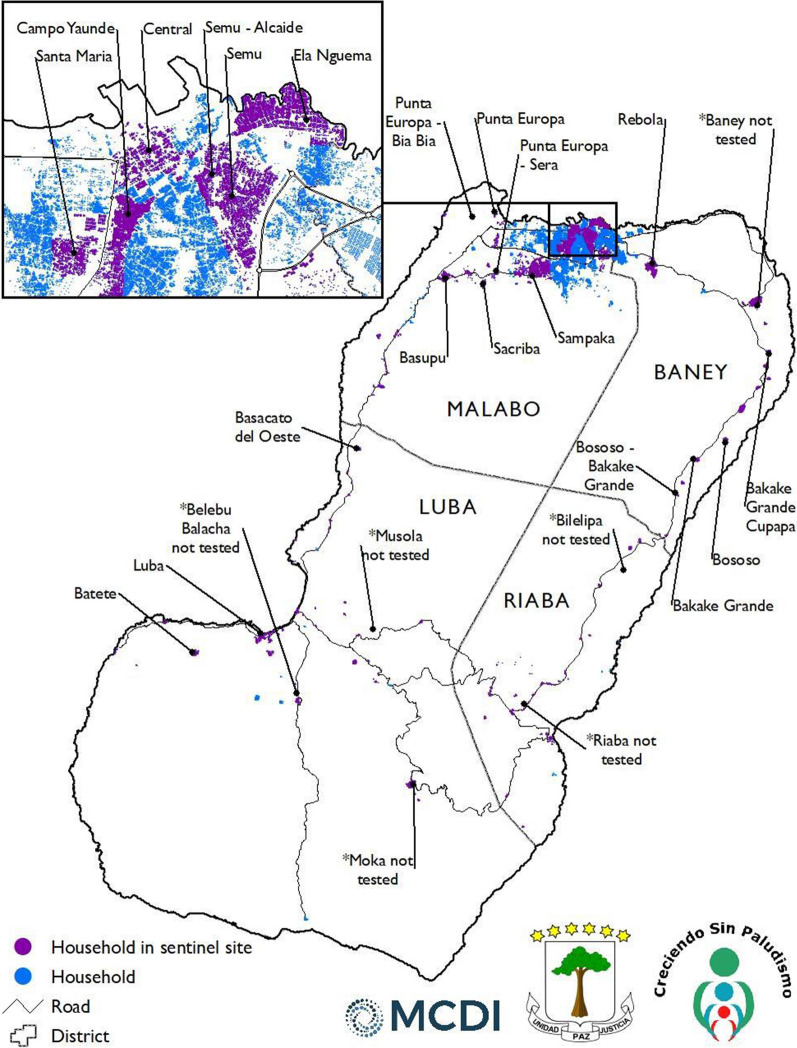
Map of Bioko Island showing historical sentinel sites for the annual Malaria Indicator Surveys under the BIMCP

At the inception of the Bioko Island Malaria Control Project (BIMCP) in 2004, island-wide indoor residual spraying (IRS) was the main bi-annual vector control activity undertaken on the island [17]. As a major community-level intervention strategy, bed nets were introduced on Bioko in late 2007 and early 2008 through a mass distribution campaign of LLINs (PermaNet 2.0^®^, Vestergaard-Frandsen) impregnated with deltamethrin. The distribution was carried out by the Equatorial Guinea National Malaria Control Programme (NMCP), with the support of the BIMCP. As such, a total of 111,301 LLINs were distributed and installed through door-to-door direct hang-up by teams of community-based volunteers under the supervision of the Spanish Red Cross. In addition, from the beginning of the project, the BIMCP has conducted annual Malaria Indicators Surveys (MIS), within 18 sentinel sites on the Island, as part of its evaluation plan to measure the impact of the project's interventions. Hence, between February 25^th^ and March 31st, 2012, MIS data was used to re-visit households that indicated having LLINs during the survey to obtain LLIN samples (Fig. 1; map showing the sentinel sites) to investigate their insecticidal activity and insecticide concentration.

These sites were selected because of the entomological monitoring and vector control activities conducted within them. Only households located within the 18 sentinel sites that had received LLINs from the BIMCP during the 2007/2008 mass distribution campaign and through antenatal care (ANC) distributions for the next four years were eligible for the sampling process. Households within the sentinel sites indicating and confirming ownership by observing their LLINs during the 2011 MIS were randomly selected using the BIMCP mapping system [17]. During the household visit to collect the nets, household residents provided the estimated age of the net, which the surveyor verified by checking the stated date of manufacture on the label of the net. A total of 356 LLINs were collected in March 2012 from 235 randomly selected households, each time replacing collected nets with new ones. Additional information was recorded at the household level and on the LLINs, including the sentinel site in which the house belonged, unique house identification number, the duration of net usage (i.e., age), net manufacturer information, net expiry date, and batch number.

**Fig. 2:**
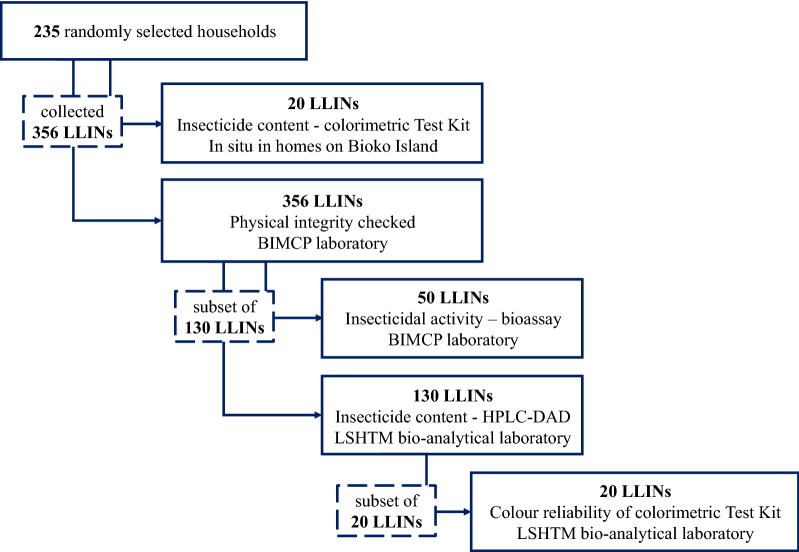
Flow diagram to show the total number of nets collected and assessed at BIMCP laboratory on Bioko Island and subset of nets selected for further analysis (insecticide content and insecticidal activity) at LSHTM and BIMCP

Following WHO guidelines, the physical integrity of the 356 PermaNet 2.0^®^ LLINs collected from 235 randomly selected households, were assessed at the BIMCP laboratory (Fig. 2). A subset of 130 LLINs were selected to additionally assess the insecticidal activity and insecticide content. At the London School of Hygiene and Tropical Medicine (LSHTM), UK, bio-analytical laboratory the insecticide content was determined for all 130 nets, while at the BIMCP laboratory the residual insecticidal activity was determined for 50 of the 130 nets.

The nets were grouped into five categories according to the duration of their use: Group 1, 0–11 months (n = 20); Group 2, 12–23 months (n = 24); Group 3, 24–35 months (n = 31); Group 4, 36–47 months (n = 28); and Group 5, 48–52 months (n = 27). A colorimetric Test Kit, designed for use as a screening tool, was also used for the first time to detect deltamethrin on LLINs deployed on Bioko Island, Equatorial Guinea. This Test Kit was applied to two subsets of randomly selected LLINs. One subset on Bioko Island to trial in situ testing in the field (20 nets from the 356 collected nets), and a second subset at LSHTM to determine the reliability of the colour produced from the kit (20 nets from the subset of 130 nets).

### Analysis of the insecticide content and insecticidal activity of LLINs

A subset of LLINs were randomly selected. Each net was divided into five sections (the 'roof,' two wide and two narrow sides) from which the insecticide content and insecticidal activity for the net as a whole was calculated. Deltamethrin content was assessed using the colorimetric Test Kit [16], and quantified using high-performance liquid chromatography with diode array detection (HPLC–DAD), while the insecticidal activity was assessed using cone bioassays [10].

#### a) High-performance liquid chromatography determination of insecticide concentration

The deltamethrin concentration was determined for all 130 LLINs in the bio-analytical laboratory at LSHTM using HPLC–DAD [18]. For each section of the LLIN (the 'roof,' two wide and two narrow sides), three squares (1 × 1 cm) were removed for analysis. The deltamethrin in the netting was extracted using acetonitrile (1 ml) under sonication for 5 min. The supernatant was then injected into the HPLC column, and the quantity of deltamethrin present was measured using Thermo Scientific™ Dionex™ Ultimate™ 3000 HPLC–DAD system (Thermofisher, Hemel Hempstead, UK) and separation was achieved using a GENESIS^®^ AQ 4 µm column (150 × 4.6 mm, Grace Materials Technologies, Cranforth, UK). A gradient of ammonium formate (10 mM, pH 2.7) and acetonitrile (v/v; 15:85 to 85:15 over 7.0 min) was used as the mobile phase, flowing at 1 mL/min through the diode array detector (UV-DAD 3000) set at 204 nm for deltamethrin. The authenticity of the detected peaks was determined by comparison of retention time, spectral extraction at 204 nm, and spiking the sample with commercially available deltamethrin (from Sigma-Aldridge, UK). A calibration curve of insecticide was generated by Chromeleon (Thermo Scientific™ Dionex™ Chromeleon™ 6.8 Chromatography Data System) using known amounts of the standard deltamethrin in acetonitrile injected onto the column. The deltamethrin concentration (mg/mL) was calculated for each net section and then averaged to provide a milligram per square metre (mg/m^2^) concentration for the net as a whole. The deltamethrin concentration across the five age groups of nets was evaluated using an unequal variances t-test and two proportion z-test.

#### b) Colorimetric analysis determination of insecticide content

A colorimetric Test Kit was used to detect deltamethrin on LLINs. This Test Kit was applied to two subsets of randomly selected LLINs, one subset on Bioko Island (20 nets) and a second subset at LSHTM (20 nets).

**Fig. 3:**
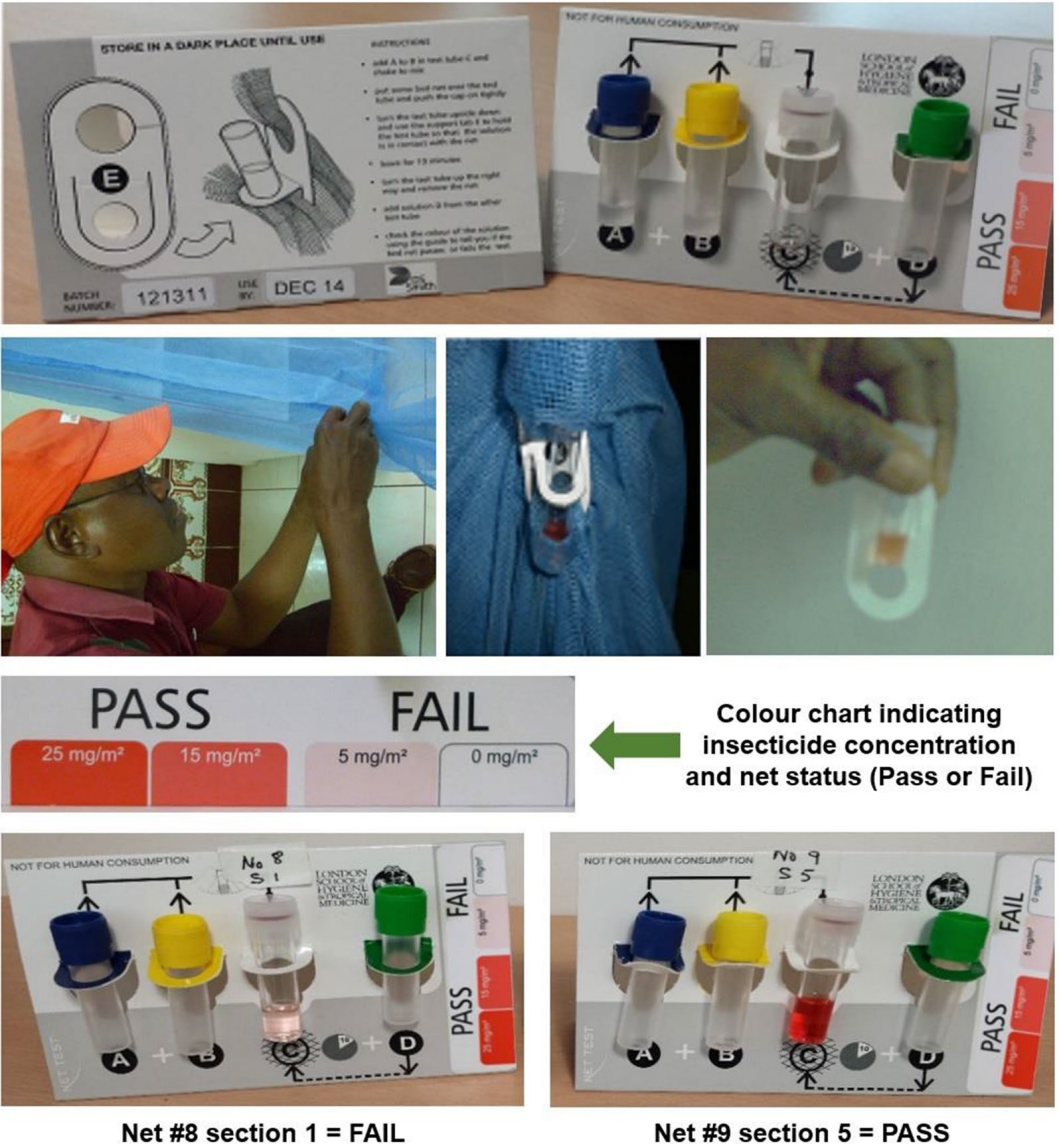
Colorimetric Test Kit in use attached to the net on Bioko Island to test the insecticide residue of LLINs in situ

The test is based on detecting cyanide ions released from type II pyrethroids and can be performed in situ. On Bioko Island, the insecticide content of the 20 LLINs was tested in situ*,* i.e., while hanging in place at a household (Fig. 3), before collection and replacement with new nets. The colorimetric test was applied to five sections for each LLIN, from which the insecticide content for the net as a whole was calculated. The surveyors on the ground found the test simple to use and appreciated the short time (< 15 min) to achieve results. The depth of colour was assessed visually by the surveyor, and no camera was used in the field.

The colorimetric Test Kit comprises of three tubes containing Reagent A, B, and D, and a fourth tube C to carry out the test. Reagent A (25 mM potassium hydroxide in analytical grade ethanol) is added to B (*para*-nitrobenzaldehyde 6.25 mM plus 2,3,5 triphenyl tetrazolium chloride 20 mM in analytical grade ethanol) in tube C and shaken to mix the two reagents. The test bed net is placed over tube C, and the cap is tightly pushed on. The tube is turned upside down, and stopper E is used to hold the tube so that the solution is in direct contact with the part of the test net.

After 10 min, the tube is turned up so that the solution is away from contact with the net. The tube is then removed from the net. Glacial acetic acid (10%) in analytical grade ethanol (solution D) is then added to stop the reaction. The resultant depth of the orange-red colour produced indicates the amount of deltamethrin detected in the test. Calibrating solutions containing known concentrations of deltamethrin (between 0 and 100 mg/mL) were used for the colorimetric Test Kit to visually determine the amount of deltamethrin detected and produce the colour chart shown on the side of the Kit. Furthermore, the colour of the net was not found to interfere with the orange-red colour produced in the test. The lack of evidence in the literature for a minimum insecricide concentration threshold required to kill the mosquito, external consultation from LSHTM vector control experts (M Rowland and J Lines, personal communication) was sought. Their advice was to use a concentration of < 15 mg/m^2^ as the cut-off value for insecticidal effectiveness of the nets against mosquitoes (“Fail” versus “Pass” on the Test Kit). More studies in different locations using the colorimetric test kits are needed to provide conclusive data to provide evidence to the control programmes regarding the cut-off as determined by the net Test Kit at which the net should be replaced as it will not kill the mosquitoes.

At LSHTM the reliability between the colour produced in the colorimetric Test Kit [16] and the deltamethrin concentration was assessed. From the 130 LLINs collected from households on Bioko Island, a subset of 20 LLINs were randomly selected across the observed range of deltamethrin concentrations. Following the instructions on the test kit, the colorimetric test was performed on each section of the LLINs following the same protocol used on Bioko Island. After 10 min, the colour reaction was stopped and recorded using a digital camera (Pentax™, Optio S7). The colour produced was quantified by measuring the red, green, and blue (RGB) colour components using the digital picture-processing program Photoshop [20]. The RGB values (0 to 255) were combined and log-transformed to determine the colour value. The colour value was then compared to the test sample's corresponding deltamethrin concentration, determined using HPLC–DAD [19], to determine the relationship between colour produced and deltamethrin concentration. A similar approach has been used in other colour reaction analyses [21].

#### c) Bioassay determination of the insecticidal activity

The bio-efficacy of the LLINs was evaluated for a subset of 50 LLINs from the 130 LLINs, representing 38% of the analysed nets. The residual insecticide activity was assessed at the BIMCP laboratory using the WHO cone tests [22] using the progeny of wild-caught and susceptible *Anopheles gambie *sensu lato female mosquitoes reared at the laboratory. Three to five-day-old unfed female mosquitoes were used for the tests. Four WHO bioassay cones were fastened to a 25 cm x 25 cm section of the net from four sides of the LLIN; the duplicate wide side of the net was excluded. The cone tests were performed twice using five F1 adult female mosquitoes per cone (10 in total per cone and section of the net), resulting in the exposure of 40 mosquitoes per bed net section tested. Mosquito mortality was recorded 24 h after exposure, based on the proportion of dead mosquitoes in relation to the total number of mosquitoes exposed. A negative control with an untreated net sample was used. The insectary had a temperature-controlled environment between 26 ± 2 °C and humidity between 75 and 90%.

## Results

The deltamethrin concentration was determined for 130 LLINs, grouped according to the duration of use, using HPLC–DAD (Additional file 1: Table S1). The deltamethrin concentration was determine for the LLIN as a whole and exhibited an overall significant decrease with net age from 65 mg/m^2^ (Group 1 = 0–11 months) to 31 mg/m^2^ (Group 5 = 48–52 months; unequal variances t-test, p < 0.001; Table 1). From the 130 nets, 18% were found to have a deltamethrin concentration < 15 mg/m^2^, i.e., nets that fall within the “Fail” category according to the colorimetric Test Kit. The proportion of nets with low deltamethrin concentrations (< 15 mg/m^2^) exhibited a significant increase with age, i.e., 5% of the nets did not meet the threshold concentration in Group 1 (0–11 months) compared to 30% in Group 5 (48–52 months; two proportion z test, p = 0.034).

**Table 1:** Average deltamethrin (DM) concentration and the proportion of LLINs that were below the threshold concentration of deltamethrin (DM < 15 mg/m^2^) for each age group of LLINs, determined using HPLC–DAD

Group	Age (months)	Total LLINs	LLINs (%) with DM < 15 mg/m^2^	Whole net DM concentration (mg/m^2^)
1	0–11	20	1 (5.0%)	65.10 ± 24.19
2	12–23	24	2 (8.3%)	48.28 ± 25.89
3	24–35	31	4 (12.9%)	42.22 ± 26.81
4	36–47	28	8 (28.6%)	43.27 ± 39.26
5	48–52	27	8 (29.6%)	30.97 ± 24.41
Total		130	23	

**Fig. 4:**
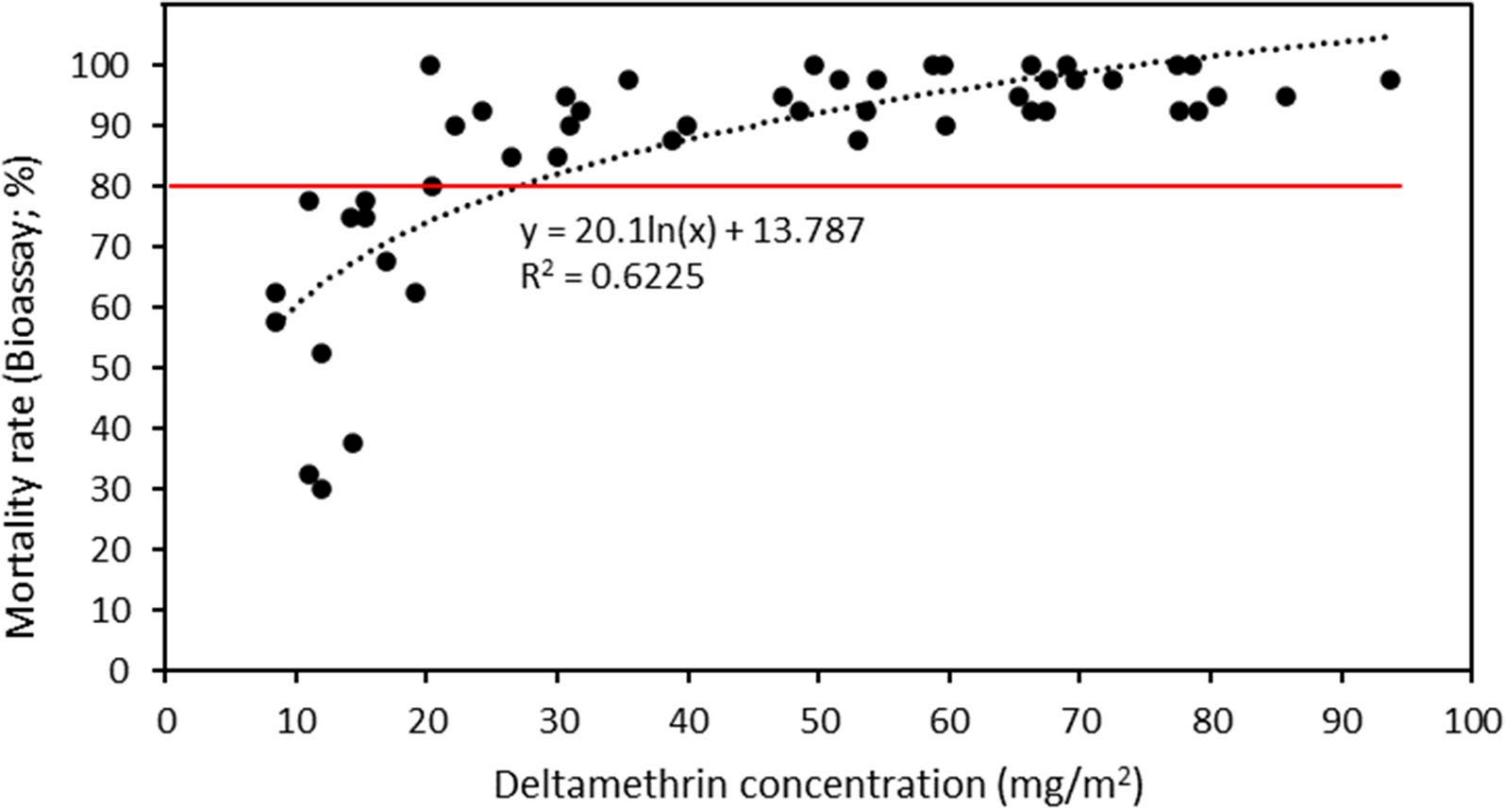
Relationship between mosquito mortality rate (%) and deltamethrin concentration (mg/m^2^). The red line indicates 80% mortality (WHO mortality cut-off)

For the subset of 50 selected LLINs, whole net bio-efficacy (average mortality of susceptible mosquitoes) was compared to the nets' overall deltamethrin concentration (Additional file 1: Table S1). The deltamethrin concentration of these nets ranged between 8 and 94 mg/m^2^ (calculated for the net as a whole), with 76% of the nets producing ≥ 80% mortality (Fig. 4 and Table 2). The mortality cut-off used follows the World Health Organization’s test procedures for insecticide resistance monitoring in malaria vector mosquitoes, where ≥ 80% of the mosquitoes are dead after 24 h [11]. Overall, there was a logarithmic relationship between mortality rate and deltamethrin concentration (y = 20.1ln(x) + 13.787; R^2^ = 0.6225). There was some variability between the bioassay and HPLC–DAD results; however, all nets with a deltamethrin concentration > 25 mg/m^2^ had ≥ 80% mortality, while all nets with a concentration < 15 mg/m^2^ had < 80% mortality. Of the eight nets with a deltamethrin concentration of 15–25 mg/m^2^, half had < 80% mortality, and the other half had ≥ 80% mortality. Nets with reduced bioassay mortality rates were found to have deltamethrin concentrations close to the 15 mg/m^2^ cut-off value and substantially higher deltamethrin concentrations on the roof sections compared to the other four sections, thus elevating the deltamethrin concentration for the net as a whole (Additional file 1: Table S1).

**Table 2:** Whole net deltamethrin (DM) concentration (mg/m^2^) and mortality rate (%) of 50 LLINs grouped according to the age of the LLINs

Sentinel site	Age (months)	DM concentration (mg/m^2^)	Test Kit category	Mortality rate (%)	Manufacture date	Batch number
Group 1 = 0–11 months						
Luba	2	93.74 ± 8.32	Pass (> 25)	97.5	Dec-07	15277
Bakake Grande	3	14.15 ± 9.83	Fail (5–15)	75	Illegible	Illegible
Santa Maria	4	30.69 ± 13.20	Pass (> 25)	95	Dec-07	15277
Central	5	53.65 ± 10.84	Pass (> 25)	92.5	Dec-07	15277
Semu	5	69.05 ± 2.93	Pass (> 25)	100	Dec-07	15277
Campo Yaunde	7	85.72 ± 5.69	Pass (> 25)	95	Dec-07	15307
Central	8	67.53 ± 20.42	Pass (> 25)	97.5	Dec-07	15307
Punta Europa–Bia Bia	9	58.76 ± 21.2	Pass (> 25)	100	Dec-07	15277
Sampaka	10	78.60 ± 7.63	Pass (> 25)	100	Jul-07	30357
Group 2 = 12–23 months						
Central	12	20.28 ± 10.29	Pass (15–25)	100	Nov-08	16498
Rebola	12	35.36 ± 21.55	Pass (> 25)	97.5	Jul-07	Illegible
Semu	12	49.58 ± 20.18	Pass (> 25)	100	Dec-07	15297
Punta Europa–Sera	14	77.61 ± 7.76	Pass (> 25)	92.5	Dec-07	15277
Bakake Grande–Cupapa	14	59.62 ± 17.14	Pass (> 25)	100	Dec-07	15307
Bakake Grande–Cupapa	14	77.40 ± 7.38	Pass (> 25)	100	Dec-07	15297
Semu–Alcaide	14	66.33 ± 8.16	Pass (> 25)	92.5	Jan-07	10197
Santa Maria	15	10.98 ± 12.35	Fail (5–15)	77.5	Dec-07	15287
Batete	15	19.09 ± 13.23	Pass (15–25)	62.5	Jul-07	30357
Santa Maria	17	65.34 ± 11.76	Pass (> 25)	95	Dec-07	15277
Punta Europa	18	24.30 ± 11.76	Pass (15–25)	92.5	Dec-07	15307
Ela Nguema	18	66.25 ± 8.95	Pass (> 25)	100	Dec-07	15277
Ela Nguema	18	15.22 ± 16.49	Pass (15–25)	75	Jul-07	30357
Group 3 = 24–35 months						
Ela Nguema	24	59.71 ± 9.16	Pass (> 25)	90	Jan-07	10197
Central	25	79.05 ± 16.55	Pass (> 25)	92.5	Dec-07	15287
Sacriba	27	39.86 ± 10.53	Pass (> 25)	90	Dec-07	15277
Campo Yaunde	27	67.36 ± 9.54	Pass (> 25)	92.5	Nov-08	16498
Campo Yaunde	28	53.04 ± 16.27	Pass (> 25)	87.5	Dec-07	15297
Semu	30	8.35 ± 8.72	Fail (5–15)	62.5	Jul-07	30357
Basacato del Oeste	30	22.18 ± 10.61	Pass (15–25)	90	Jan-07	10197
Santa Maria	30	30.04 ± 13.60	Pass (> 25)	85	Jul-07	30357
Santa Maria	32	20.45 ± 5.16	Pass (15–25)	80	Mar-09	12009
Santa Maria	33	80.53 ± 9.32	Pass (> 25)	95	Dec-07	15277
Group 4 = 36–47 months						
Sampaka	36	26.40 ± 5.05	Pass (> 25)	85	Jan-07	10197
Bososo	36	31.68 ± 11.33	Pass (> 25)	92.5	Jul-07	30357
Sampaka	36	69.54 ± 6.30	Pass (> 25)	97.5	Jul-07	30357
Basopu	37	14.29 ± 9.90	Fail (5–15)	37.5	Nov-06	13646
Basopu	37	38.80 ± 10.04	Pass (> 25)	87.5	Jul-07	30357
Ela Nguema	38	47.24 ± 6.13	Pass (> 25)	95	Jan-07	10197
Punta Europa	40	11.90 ± 10.83	Fail (5–15)	30	Jul-07	30357
Bososo–Bakake Grande	41	8.39 ± 5.45	Fail (5–15)	57.5	Jul-07	30357
Bososo–Bakake Grande	41	16.95 ± 5.35	Pass (15–25)	67.5	Jul-07	Illegible
Group 5 = 48–52 months						
Semu	48	54.38 ± 9.33	Pass (> 25)	97.5	Dec-07	15277
Semu–Alcaide	48	48.45 ± 9.82	Pass (> 25)	92.5	Dec-07	15277
Punta Europa	48	11.89 ± 9.64	Fail (5–15)	52.5	Jan-07	10197
Semu–Alcaide	48	34.41 ± 17.15	Pass (> 25)	97.5	Jul-07	30357
Rebola	50	72.43 ± 8.90	Pass (> 25)	97.5	Jul-07	30357
Basopu	50	30.91 ± 6.20	Pass (> 25)	90	Jul-07	30357
Campo Yaunde	50	10.97 ± 4.86	Fail (5–15)	32.5	Jan-07	10197
Campo Yaunde	50	51.64 ± 12.76	Pass (> 25)	97.5	Jul-07	30357
Ela Nguema	51	22.42 ± 18.51	Pass (15–25)	77.5	Jul-07	30357

**Fig. 5:**
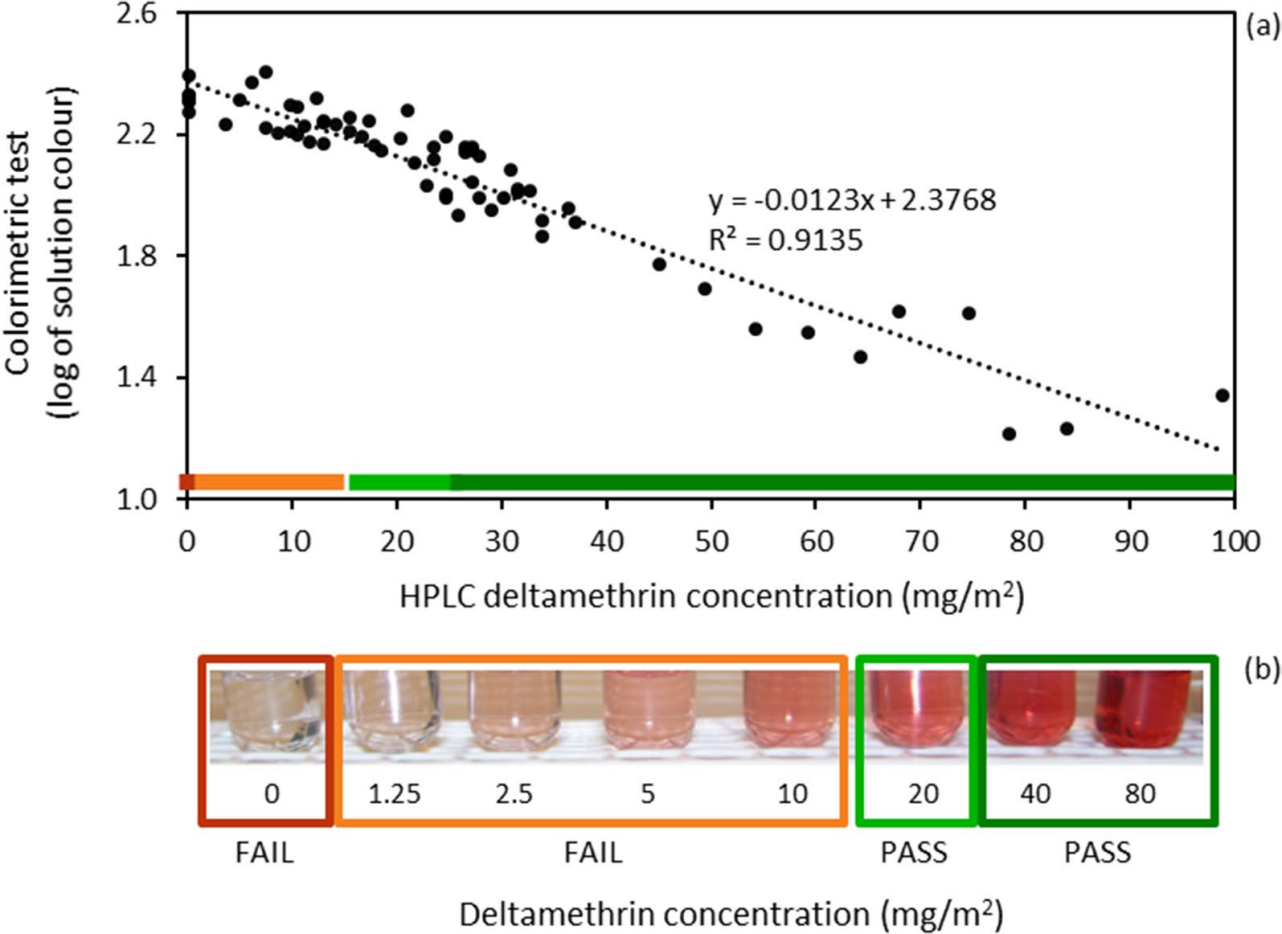
**a** Relationship between depth of colour (log-transformed) produced, using the colorimetric Test Kit, and the deltamethrin concentration (mg/m^2^), determined using HPLC–DAD, for 20 LLINs; and **b** depth of colour range exhibited using the colorimetric Test Kit across a range of deltamethrin concentrations from 0 to 80 mg/m^2^. In Fig. **a**, the orange line (dark and light) indicates nets that fell into the “Fail” category of the colorimetric test (< 15 mg/m^2^), while the green line (light and dark) indicates nets that fell into the “Pass” category

The reliability of the colorimetric Test Kit was assessed using a digital camera to determine the colour produced, quatified using the RGB colour components, across a range of deltamethrin concentrations (0.1 to 100 mg/m^2^). For each section of net tested the depth of the orange-red colour produced was compared to its corresponding deltamethrin concentration (20 LLINs, five sections per net). The comparison showed that there was a linear relationship between the colour produced and the deltamethrin concentration observed (y = − 0.0123x + 2.3768; R^2^ = 0.9135; Fig. 5.

## Discussion

An LLIN is a factory-treated net with insecticide either incorporated into or coated around the fibres, expected to retain its biological activity for a minimum of 20 standard WHO washes under laboratory conditions and three years of recommended use under field conditions [23]. The rate at which insecticide activity wears off over time is influenced by wear and tear during use and the surrounding environmental conditions. NMCPs distribute nets and implement spray operations as primary malaria vector control interventions; hence, monitoring tools to decision making at the programme level are essential for providing information on the quality of spray performance or when nets need to be replaced after a distribution cycle. Generally, monitoring the durability of LLINs is restricted to reports from the manufacturers, health staff, questioning the net users, and carrying out durability monitoring studies. Moreover, measuring the amount of insecticide on the net requires the use of confirmatory laboratory methods based on gas or liquid chromatography, screening field colorimetric methods, or indirectly by cone bioassays [24].

Colorimetric field tests have been a practical and effective way to monitor and measure the insecticide concentration [24] on PermaNet 2.0 (PowerNets™) LLINs in rural Lao PDR [25]. The study in Lao also used HPLC and mosquito bioassays, and results showed that less than 10% of deltamethrin was found on the nets after 24 months of use, which is a significant finding as it falls far away from the expected 3-year useful durability of the net. In a similar study in Vanuatu, colorimetric test kits were used to estimate the levels of λ-cyhalothrin, used for indoor residual spraying, and conduct overall quality control of the spray operations [22]. In both settings, the colorimetric Test Kit provided rapid and reliable information on the residual insecticidal durability of bed nets and spray performance, showcasing the test's utility during field activities to monitor vector control activities and aid the decision-making of NMCP’s to improve the efficacy of vector control operations.

In 2012, the simple, non-destructive and inexpensive colorimetric Test Kits [16] were tested on LLINs distributed on Bioko Island from a mass distribution campaign in 2007. The surveyors using the kits expressed their approval as being field-friendly, less labour intensive than cone assays, and able to do them on-site with minimal training and without the need of a laboratory. Furthermore, the field team quickly and visually interpreted results indicating the amount of deltamethrin within the sampled net within 15 min of sampling the net. To ensure the accuracy of the colorimetric test, a subset of nets with a known deltamethrin concentration (HPLC–DAD), ranging from 0.1 to 100 mg/m^2^, was also visually evaluated by the field surveyors. The depth of colour produced from the colorimetric test was found to have a linear correlation with deltamethrin concentration (y = − 0.0123x + 2.3768; R^2^ = 0.9135). The strong correlation between the solution’s depth of colour and the deltamethrin concentration shows that the colour produced by this colorimetric Test Kit is reliable. In addition to providing quick and simple assessments of insecticidal concentration, the resulting orange-red solution can be kept for > 2 months at 25 °C, as long as the stopper is secured on the tube to prevent the solution from evaporating over time, which is useful for future reference and quality control.

The LLINs analysed for this study on Bioko were stated to be manufactured in 2007. Their average deltamethrin concentration exhibited an overall significant decrease with increased age from 65 mg/m^2^, for nets used for less than 12 months, to 31 mg/m^2^, for nets used over 48 months (p < 0.001). The decrease in insecticide concentration indicates a loss of approximately 10 mg/m^2^ of deltamethrin per year of use. Insecticidal activity (bioassay mortality rate) and deltamethrin concentration (HPLC–DAD) showed a logarithmic relationship (y = 20.1ln(x) + 13.787; R^2^ = 0.6225), and a cut-off concentration of 15 mg/m^2^ in order to achieve at least 80% mortality. Across these households on Bioko, 18% of nets being used were found to have < 15 mg/m^2^ of deltamethrin thus rendering them less effective. A significant increase in nets falling below the established threshhold in line with increasing net age (< 15 mg/m^2^ of deltamethrin concentration) was also detected, from 5% of nets in the first 12 months of use to 30% of nets that have been used for more than 48 months (p = 0.034). The significant correlation between net use, i.e. age, and deltamethrin concentrations below the established threshold shows that it is vital to regularly test the deltamethrin concentration of LLINs currently in use to determine their insecticidal efficacy.

For all LLINs being procured and delivered in endemic malaria countries, there is currently no alternative insecticide to treat the nets in circulation. Pyrethroid insecticides remain effective and are used in all WHO prequalified LLINs. Recent data from Papua New Guinea has also shown that just 17% of LLINs, with manufacturing dates prior to 2013, were fulfilling the WHO bioefficacy standards, suggesting their contribution to the resurgence of malaria in the country and warranting increased scrutiny of LLINs [27]. Although synergists such as piperonyl butoxide (PBO), have come into the market there remains a need to quickly and effectively monitor the residual concentration of insecticides to aid national Programmes in planning and implementing vector control interventions. Overall, the colorimetric Test Kit is useful for all type II pyrethroids but not type I pyrethroids like permethrin as it depends on the detection of the cyanide group on the molecule, which is not present on the molecule of the synergist, PBO or permethrin. Since the implementation of this study, similar field-friendly colorimetric methods have been developed but not validated in the field, to detect type I pyrethroids [26] in any suitable object, like mosquito nets, treated with such insecticides, thus increasing the ability for such tests to be used for operational decision making. Nonetheless, further studies using the test kit must be conducted at varying locations in the malaria-endemic world where type I and II pyrethroids are deployed to provide conclusive data, that can be used for operational decision making. More importantly, additional evidence on the insecticide concentration cut-off where the net is no longer killing mosquitoes is also needed to better inform NMCPs. The design of the colorimetric Test Kit described here has the potential to be improved based on feedback from local surveyors after it being tested in more locations, leading to a commercially available product.

## Conclusion

NMCPs need access to adequate and affordable monitoring tools that can be easily used in situ by all types of staff working in vector control. Thus, providing results for managers to improve the efficacy of vector control operations. This simple, non-destructive, field-friendly and inexpensive colorimetric Test Kit was developed to detect type II pyrethroids (deltamethrin; α-cypermethrin and λ-cyhalothrin) and field evaluated on deltamethrin LLINs distributed on Bioko Island. The test takes approximately 15 min to easily interpret results visually by field personnel not experienced in laboratory methods. In addition, the test provided quick and straightforward assessments of insecticidal concentration, thus making it a valuable tool to enable control programmers to decide when the bed net needs to be replaced.

## Supplementary Information

**Additional file 1: Table S1.** Deltamethrin (DM) concentration (mg/m^2^) of 130 LLINs and mortality rate (%) of a subset of 50 LLINs grouped according to the age of the LLINs.

## Data Availability

All data are included in the manuscript and Additional file 1: Table S1.
