# Supplementary material for: A colorimetric test for the evaluation of the insecticide content of LLINs used on Bioko Island, Equatorial Guinea
Source: Malar J. 2021 Nov 10;20:433. doi: 10.1186/s12936-021-03967-w (PMC8579635; doi:10.1186/s12936-021-03967-w)
Supplement: Supplementary file 1 — Additional file 1: Table S1. Deltamethrin (DM) concentration (mg/m2) of 130 LLINs and mortality rate (%) of a subset of 50 LLINs grouped according to the age of the LLINs. [file 12936_2021_3967_MOESM1_ESM.pdf]

Table S1: Deltamethrin (DM) concentration (mg/m^2) of 130 LLINs and mortality rate (%) of a subset of 50 LLINs grouped acc

Group	Sentinel site	House ID	LLIN information: Age (months) at collection	Manufacture date	Batch number	Net sections	DM concentration (mg/m^2): Each section	Whole net
Group 1 = 0 - 11 months	Sampaka	M509S28E93P01	1	Dec-07	15277	i	76.34 ± 6.54	
						ii	90.33 ± 3.04	
						iii	82.72 ± 11.13	
						iv	82.30 ± 3.97	
						v	87.86 ± 0.36	
							Average	83.91 ± 7.26
Group 1 = 0 - 11 months	Santa Maria	M335S92E38P01	1	Dec-07	15277	i	82.72 ± 1.63	
						ii	78.81 ± 2.34	
						iii	72.84 ± 5.89	
						iv	83.74 ± 11.45	
						v	82.30 ± 2.34	
							Average	80.08 ± 6.53
Group 1 = 0 - 11 months	Central	M276S69E13P01	2	Dec-07	15277	i	75.31 ± 3.27	
						ii	67.90 ± 1.63	
						iii	77.78 ± 4.94	
						iv	72.63 ± 3.56	
						v	64.20 ± 12.14	
							Average	71.56 ± 7.37
Group 1 = 0 - 11 months	Luba	M2258S76E18P01	2	Dec-07	15277	i	94.86 ± 7.23	
						ii	101.23 ± 0	
						iii	81.07 ± 5.25	
						iv	91.98 ± 3.75	
						v	99.59 ± 2.78	
							Average	93.74 ± 8.32
Group 1 = 0 - 11 months	Bakake Grande	M1292S52E5P01	3	Ilegible	Ilegible	i	6.17 ± 1.23	
						ii	5.35 ± 0.71	
						iii	31.07 ± 2.49	

Group 1 = 0 - 11 months	Bakake Grande	[illegible]	[illegible]	Illegible	Illegible	iv	11.52 ± 0.36	
						v	16.67 ± 2.69	
							Average	14.16 ± 9.83
Group 1 = 0 - 11 months	Sampaka	M509S26E56P01	4	Dec-07	15307	i	73.05 ± 5.03	
						ii	80.25 ± 2.83	
						iii	71.19 ± 6.77	
						iv	107.41 ± 10.31	
						v	71.19 ± 11.08	
							Average	80.62 ± 15.75
Group 1 = 0 - 11 months	Santa Maria	M394S13E3P01	4	Dec-07	15277	i	30.25 ± 2.14	
						ii	17.49 ± 0.71	
						iii	53.91 ± 4.34	
						iv	21.6 ± 1.23	
						v	30.25 ± 1.23	
							Average	30.7 ± 13.21
Group 1 = 0 - 11 months	Campo Yaunde	M355S49E59P01	5	Nov-09	19329	i	27.37 ± 2.85	
						ii	11.11 ± 2.14	
						iii	41.98 ± 1.07	
						iv	94.24 ± 1.28	
						v	70.78 ± 4.11	
							Average	49.09 ± 31.04
Group 1 = 0 - 11 months	Central	M276S69E114P01	5	Dec-07	15277	i	63.08 ± 2.74	
						ii	34.06 ± 0.29	
						iii	58.24 ± 3.75	
						iv	52.82 ± 1.4	
						v	60.08 ± 2.6	
							Average	53.66 ± 10.84
Group 1 = 0 - 11 months	Semu	M278S91E26p01	5	Dec-07	15277	i	68.72 ± 1.98	
						ii	69.14 ± 3.44	
						iii	72.22 ± 2.23	
						iv	69.34 ± 2.78	
						v	65.84 ± 0.94	
							Average	69.05 ± 2.93

Group 1 = 0 - 11 months	Ela Nguema	M279S37E28P01	6	Jul-07	30357	i	80.66 ± 5.83	
						ii	89.51 ± 2.23	
						iii	88.68 ± 11.05	
						iv	79.84 ± 3.77	
						v	89.09 ± 10.64	
							Average	85.56 ± 7.84
Group 1 = 0 - 11 months	Campo Yaunde	M335S49E59P01	7	Dec-07	15307	i	80.86 ± 5.89	
						ii	85.19 ± 5.49	
						iii	81.48 ± 4.9	
						iv	91.15 ± 1.55	
						v	89.92 ± 1.55	
							Average	85.72 ± 5.7
Group 1 = 0 - 11 months	Central	M335S8E43P01	8	Jan-07	10197	i	49.38 ± 5.38	
						ii	41.15 ± 1.55	
						iii	62.76 ± 0.94	
						iv	54.73 ± 0.94	
						v	50.41 ± 8.99	
							Average	51.69 ± 8.36
Group 1 = 0 - 11 months	Central	M276S68E34P01	8	Dec-07	15307	i	36.42 ± 2.69	
						ii	81.07 ± 5.18	
						iii	58.23 ± 2.49	
						iv	68.52 ± 2.69	
						v	93.42 ± 3.17	
							Average	67.53 ± 20.43
Group 1 = 0 - 11 months	Punta Europa - Bia Bia	M210S000E67P01	9	Dec-07	15277	i	49.38 ± 3.27	
						ii	23.87 ± 3.4	
						iii	75.31 ± 3.7	
						iv	73.46 ± 12.33	
						v	71.81 ± 3.4	
							Average	58.77 ± 21.21
Group 1 = 0 - 11 months	Batete	M2252S66E8P01	10	Dec-07	15307	i	4.12 ± 4.34	
						ii	20.37 ± 2.23	
						iii	57.2 ± 3.4	

Group 1 = 0 - 11 months	Batete	M2252S66E8P01	10	Dec-07	15307	iv	44.24 ± 2.78	
						v	45.27 ± 2.92	
							Average	34.24 ± 20.09
Group 1 = 0 - 11 months	Batete	M2252S76E14P01	10	Dec-07	15277	i	57.61 ± 1.55	
						ii	61.93 ± 6.18	
						iii	74.49 ± 16.22	
						iv	80.86 ± 5.95	
						v	75.72 ± 6.66	
							Average	70.12 ± 11.76
Group 1 = 0 - 11 months	Bilelipa	M2224S66E1P01	10	Dec-07	15307	i	75.1 ± 8.95	
						ii	91.15 ± 6.54	
						iii	75.51 ± 3.17	
						iv	87.86 ± 4.47	
						v	83.33 ± 10.16	
							Average	82.59 ± 9.01
Group 1 = 0 - 11 months	Ela Nguema	M278S17E50P01	10	Jul-07	30357	i	37.65 ± 8.02	
						ii	46.91 ± 1.85	
						iii	70.78 ± 3.92	
						iv	73.87 ± 1.43	
						v	74.28 ± 2.49	
							Average	60.7 ± 16.31
Group 1 = 0 - 11 months	Sampaka	M509S17E10P01	10	Jul-07	30357	i	82.92 ± 4.11	
						ii	72.63 ± 12.95	
						iii	73.25 ± 5.39	
						iv	84.57 ± 1.63	
						v	79.63 ± 3.44	
							Average	78.6 ± 7.63
Group 2 = 12 - 23 months	Central	M277S52E101P01	12	Nov-08	16498	i	24.28 ± 3.72	
						ii	5.14 ± 0.94	
						iii	13.37 ± 2.57	
						iv	32.3 ± 3.04	
						v	26.34 ± 0.94	
							Average	20.29 ± 10.3

Group 2 = 12 - 23 months	Rebola	M459S76E6P01	12	Jul-07	Ilegible	i	13.95 ± 3.74	
						ii	17.08 ± 3.04	
						iii	68.72 ± 5.39	
						iv	44.86 ± 0.94	
						v	46.5 ± 3.17	
							Average	35.37 ± 21.55
Group 2 = 12 - 23 months	Santa Maria	M394S11E25P01	12	Nov-08	16498	i	25.51 ± 3.56	
						ii	58.23 ± 2.34	
						iii	66.05 ± 4.32	
						iv	86.83 ± 10.28	
						v	42.39 ± 0.94	
							Average	55.8 ± 22.04
Group 2 = 12 - 23 months	Semu	M278S82E29P01	12	Jul-12	15297	i	37.86 ± 4.2	
						ii	18.93 ± 2.17	
						iii	65.84 ± 2.85	
						iv	53.29 ± 5.6	
						v	72.02 ± 1.43	
							Average	49.59 ± 20.18
Group 2 = 12 - 23 months	Bakake Grande - Cupapa	M882S47E25P01	14	Dec-07	15307	i	59.88 ± 0.62	
						ii	32.72 ± 0.62	
						iii	52.06 ± 1.55	
						iv	76.75 ± 1.43	
						v	76.75 ± 1.28	
							Average	59.63 ± 17.15
Group 2 = 12 - 23 months	Bakake Grande - Cupapa	M882S47E25P01	14	Dec-07	15297	i	68.72 ± 4.95	
						ii	75.72 ± 4.95	
						iii	75.72 ± 2.17	
						iv	88.07 ± 4.47	
						v	78.81 ± 3.56	
							Average	77.41 ± 7.38
Group 2 = 12 - 23 months	Campo Yaunde	M335S39E72P01	14	Dec-07	15277	i	51.23 ± 4.82	
						ii	41.15 ± 5.18	
						iii	63.37 ± 2.49	

Group 2 = 12 - 23 months	Campo Yaunde	[illegible]	14	Dec-07	15277	iv	60.29 ± 1.55	
						v	58.02 ± 5.89	
							Average	54.81 ± 8.97
Group 2 = 12 - 23 months	Central	M277S51E2P01	14	Dec-07	15297	i	18.93 ± 0.71	
						ii	27.57 ± 8.87	
						iii	79.63 ± 5.66	
						iv	64.61 ± 3.4	
						v	72.63 ± 3.92	
							Average	52.67 ± 25.89
Group 2 = 12 - 23 months	Punta Europa - Sera	M447S52E10P01	14	Dec-07	15277	i	79.72 ± 3.75	
						ii	66.24 ± 3.17	
						iii	79.34 ± 3	
						iv	77.6 ± 6.37	
						v	85.16 ± 7.07	
							Average	77.61 ± 7.77
Group 2 = 12 - 23 months	Semu - Alcaide	M278S92E80P01	14	Jan-07	10197	i	67.49 ± 6.31	
						ii	65.02 ± 4.11	
						iii	54.73 ± 5.25	
						iv	68.11 ± 1.43	
						v	76.34 ± 4.34	
							Average	66.34 ± 8.16
Group 2 = 12 - 23 months	Bakake Grande	M1292S43E11P01	15	Jul-07	30357	i	68.93 ± 2.34	
						ii	76.54 ± 3.44	
						iii	74.28 ± 2.92	
						iv	78.19 ± 3.97	
						v	81.69 ± 1.89	
							Average	75.93 ± 5.08
Group 2 = 12 - 23 months	Batete	M2252S76E10P01	15	Jul-07	30357	i	0	
						ii	10.08 ± 1.28	
						iii	36.42 ± 3.27	
						iv	23.25 ± 1.28	
						v	25.72 ± 0.94	
							Average	19.09 ± 13.23

Group 2 = 12 - 23 months	Santa Maria	M335S53E19P01	15	Dec-07	15287	i	0	
						ii	0	
						iii	32.51 ± 1.98	
						iv	10.49 ± 2.23	
						v	11.93 ± 0.36	
							Average	10.99 ± 12.35
Group 2 = 12 - 23 months	Sampaka	M509S52E18P01	17	Dec-07	15307	i	0	
						ii	29.84 ± 7.47	
						iii	61.93 ± 4.2	
						iv	38.07 ± 0.94	
						v	41.56 ± 3.77	
							Average	34.28 ± 21.14
Group 2 = 12 - 23 months	Santa Maria	M333S93E53P01	17	Dec-07	15277	i	63.17 ± 3.51	
						ii	46.09 ± 2.57	
						iii	79.63 ± 1.63	
						iv	66.67 ± 4.32	
						v	71.19 ± 2.34	
							Average	65.35 ± 11.77
Group 2 = 12 - 23 months	Ela Nguema	M219S85E57P01	18	Dec-07	15277	i	18.93 ± 4.55	
						ii	70.16 ± 3.51	
						iii	45.06 ± 10.2	
						iv	80.25 ± 6.68	
						v	71.6 ± 1.63	
							Average	57.2 ± 23.79
Group 2 = 12 - 23 months	Ela Nguema	M279S11E9P01	18	Jul-07	30357	i	13.17 ± 0.94	
						ii	0	
						iii	42.8 ± 2.49	
						iv	0	
						v	20.16 ± 4.34	
							Average	15.23 ± 16.5
Group 2 = 12 - 23 months	Ela Nguema	M219S85E72P01	18	Dec-07	15277	i	54.32 ± 3.21	
						ii	60.29 ± 1.28	
						iii	68.93 ± 7.21	

Group 2 = 12 - 23 months	Ela Nguema	[illegible]	18	Dec-07	15277	iv	74.28 ± 6.48	
						v	73.46 ± 1.63	
							Average	66.26 ± 8.95
Group 2 = 12 - 23 months	Punta Europa	M211S27E5P01	18	Dec-07	15307	i	8.92 ± 1.48	
						ii	34.98 ± 1.89	
						iii	33.88 ± 0.92	
						iv	23.86 ± 3.03	
						v	39.56 ± 2.75	
							Average	27.89 ± 11.57
Group 2 = 12 - 23 months	Sampaka	M509S28E93P01	18	Dec-07	15307	i	22.35 ± 19.51	
						ii	10.49 ± 1.85	
						iii	24.69 ± 2.69	
						iv	34.77 ± 3.04	
						v	12.72 ± 5.27	
							Average	20.27 ± 12.82
Group 2 = 12 - 23 months	Semu	M277S71E88P01	18	Dec-07	15277	i	6.38 ± 0.36	
						ii	8.85 ± 0.94	
						iii	24.28 ± 3.11	
						iv	9.67 ± 3.72	
						v	25.31 ± 1.63	
							Average	14.9 ± 8.67
Group 2 = 12 - 23 months	Central	M276S88E22P01	19	Jan-07	10197	i	62.55 ± 2.17	
						ii	62.96 ± 2.69	
						iii	40.74 ± 5.05	
						iv	63.99 ± 4.11	
						v	64.2 ± 7.71	
							Average	58.89 ± 10.24
Group 2 = 12 - 23 months	Basacato del Oeste	M1321S37E10P01	20	Jan-07	10197	i	72.02 ± 1.28	
						ii	66.46 ± 6.18	
						iii	47.12 ± 3.4	
						iv	71.19 ± 6.18	
						v	72.02 ± 0.94	
							Average	65.76 ± 10.52

Group 2 = 12 - 23 months	Basacato del Oeste	M1321S37E10P01	20	Jan-07	10197	i	72.02 ± 2.78	
						ii	73.87 ± 0.94	
						iii	70.16 ± 5.7	
						iv	75.31 ± 3.44	
						v	75.31 ± 2.83	
							Average	73.33 ± 3.6
Group 3 = 24 - 35 months	Ela Nguema	M279S12E45P01	24	Jan-07	10197	i	62.14 ± 2.78	
						ii	57.2 ± 0.94	
						iii	47.12 ± 3.11	
						iv	72.22 ± 5.89	
						v	59.88 ± 6.68	
							Average	59.71 ± 9.17
Group 3 = 24 - 35 months	Rebola	M460S33E9P01	24	Jul-07	30357	i	14.61 ± 1.89	
						ii	15.64 ± 2.49	
						iii	19.96 ± 4.79	
						iv	10.86 ± 3.51	
						v	24.9 ± 4.55	
							Average	16.45 ± 6
Group 3 = 24 - 35 months	Rebola	M460S83E9P01	24	Jul-07	30357	i	61.11 ± 9.88	
						ii	76.34 ± 3.17	
						iii	71.6 ± 3.75	
						iv	70.58 ± 3.62	
						v	82.72 ± 3.21	
							Average	72.47 ± 8.66
Group 3 = 24 - 35 months	Riaba	M2694S63E13P01	24	Apr-06	15277	i	58.02 ± 3.21	
						ii	66.87 ± 7.62	
						iii	82.92 ± 3.17	
						iv	56.79 ± 1.85	
						v	59.26 ± 2.83	
							Average	64.77 ± 10.69
Group 3 = 24 - 35 months	Riaba	M2694S53E4P01	24	Nov-08	16498	i	76.54 ± 3.09	
						ii	95.88 ± 2.85	
						iii	66.46 ± 2.17	

Group 3 = 24 - 35 months	Riaba	[illegible]	24	Nov-08	16498	iv	98.15 ± 6.26	
						v	91.15 ± 6.31	
							Average	85.64 ± 13.17
Group 3 = 24 - 35 months	Santa Maria	M394S1E38P01	24	Dec-07	15297	i	43.21 ± 0.62	
						ii	28.6 ± 1.78	
						iii	47.74 ± 3.62	
						iv	48.35 ± 0.36	
						v	52.67 ± 2.92	
							Average	44.12 ± 8.82
Group 3 = 24 - 35 months	Santa Maria	M394S1E38P01	24	Dec-07	15307	i	61.52 ± 1.78	
						ii	53.91 ± 3.51	
						iii	69.96 ± 1.89	
						iv	60.7 ± 1.98	
						v	57.41 ± 2.47	
							Average	60.7 ± 5.91
Group 3 = 24 - 35 months	Central	M277S53E1P01	25	Dec-07	15287	i	79.42 ± 11.13	
						ii	88.89 ± 6.96	
						iii	51.44 ± 1.98	
						iv	95.06 ± 7.48	
						v	80.45 ± 3.11	
							Average	79.05 ± 16.55
Group 3 = 24 - 35 months	Sampaka	M509S28E93P01	26	Dec-07	15287	i	39.3 ± 8.91	
						ii	59.05 ± 4.38	
						iii	74.69 ± 4.9	
						iv	82.51 ± 2.49	
						v	86.21 ± 1.28	
							Average	68.35 ± 18.38
Group 3 = 24 - 35 months	Campo Yaunde	M335S59E32P01	27	Nov-08	16498	i	50 ± 1.23	
						ii	70.16 ± 0.71	
						iii	69.75 ± 2.69	
						iv	73.87 ± 4.67	
						v	73.05 ± 4.67	
							Average	67.37 ± 9.54

Group 3 = 24 - 35 months	Campo Yaunde	M335S59E1P01	27	Jan-07	10197	i	25.93 ± 5.66	
						ii	14.94 ± 4.22	
						iii	50.21 ± 2.78	
						iv	47.12 ± 1.43	
						v	55.35 ± 3.72	
							Average	35.91 ± 17.33
Group 3 = 24 - 35 months	Campo Yaunde	M335S28E56P01	27	Jan-08	10198	i	16.46 ± 1.28	
						ii	30.45 ± 0.36	
						iii	41.56 ± 3.4	
						iv	37.24 ± 4.67	
						v	25.93 ± 2.47	
							Average	30.33 ± 9.41
Group 3 = 24 - 35 months	Sacriba	M502S65E3P01	27	Dec-07	15277	i	40.12 ± 6.44	
						ii	29.75 ± 10.21	
						iii	43.42 ± 1.43	
						iv	40.33 ± 2.78	
						v	52.47 ± 10.71	
							Average	39.87 ± 10.53
Group 3 = 24 - 35 months	Campo Yaunde	M335S68E80P01	28	Dec-07	15297	i	41.98 ± 2.69	
						ii	28.19 ± 3.4	
						iii	69.96 ± 2.34	
						iv	63.37 ± 3.4	
						v	61.73 ± 2.23	
							Average	53.05 ± 16.27
Group 3 = 24 - 35 months	Campo Yaunde	M335S49E62P01	28	Jan-07	10197	i	10.08 ± 0.94	
						ii	24.69 ± 3.21	
						iii	62.14 ± 3.62	
						iv	40.53 ± 0.94	
						v	33.95 ± 1.23	
							Average	34.28 ± 17.99
Group 3 = 24 - 35 months	Central	M335S8E43P01	28	Nov-08	16498	i	52.26 ± 1.43	
						ii	47.94 ± 4.11	
						iii	7.41 ± 3.27	

Group 3 = 24 - 35 months	Central	[illegible]	28	Nov-08	10498	iv	59.67 ± 5.6	
						v	45.68 ± 2.69	
							Average	42.59 ± 19.12
Group 3 = 24 - 35 months	Ela Nguema	M278S7E109P01	28	Dec-07	15287	i	53.7 ± 4.32	
						ii	67.7 ± 0.36	
						iii	77.57 ± 6.31	
						iv	70.16 ± 0.94	
						v	70.99 ± 2.83	
							Average	68.02 ± 8.72
Group 3 = 24 - 35 months	Santa Maria	M334S90E62P01	28	Dec-07	15297	i	33.95 ± 3.44	
						ii	47.74 ± 1.78	
						iii	65.02 ± 0.71	
						iv	73.05 ± 8.56	
						v	79.63 ± 6.42	
							Average	59.88 ± 17.91
Group 3 = 24 - 35 months	Semu	M277S71E01P01	28	Jan-07	10197	i	0	
						ii	0	
						iii	19.34 ± 1.43	
						iv	31.69 ± 3.77	
						v	36.01 ± 4.99	
							Average	17.41 ± 15.95
Group 3 = 24 - 35 months	Sampaka	M509S7E54P01	29	Jan-07	10197	i	8.44 ± 1.28	
						ii	0	
						iii	16.46 ± 4.11	
						iv	9.26 ± 0.62	
						v	15.84 ± 4.95	
							Average	10 ± 6.67
Group 3 = 24 - 35 months	Sampaka	M509S7E54P01	29	Ilegible	Ilegible	i	18.11 ± 2.34	
						ii	0	
						iii	15.43 ± 2.23	
						iv	15.43 ± 2.69	
						v	33.74 ± 5.74	
							Average	16.54 ± 11.42

Group 3 = 24 - 35 months	Sampaka	M509S28E86P01	29	Jan-07	10197	i	20.78 ± 1.43	
						ii	27.78 ± 4.28	
						iii	31.69 ± 4.99	
						iv	27.78 ± 0	
						v	32.92 ± 4.55	
							Average	28.19 ± 5.35
Group 3 = 24 - 35 months	Basacato del Oeste	M1321S27E6P01	30	Jan-07	10197	i	20.99 ± 4.28	
						ii	19.55 ± 0.36	
						iii	19.34 ± 6	
						iv	26.54 ± 1.28	
						v	25.1 ± 0.36	
							Average	22.3 ± 4.38
Group 3 = 24 - 35 months	Basacato del Oeste	M1321S27E6P01	30	no tiene	No tiene	i	0	
						ii	0	
						iii	0	
						iv	0	
						v	0	
							Average	0
Group 3 = 24 - 35 months	Central	M276S60E83P02	30	Jan-07	10197	i	15.43 ± 8.3	
						ii	17.28 ± 2.69	
						iii	17.7 ± 0.36	
						iv	16.26 ± 1.89	
						v	13.99 ± 3.77	
							Average	16.13 ± 3.92
Group 3 = 24 - 35 months	Santa Maria	M335S95E35P01	30	Jul-07	30357	i	15.23 ± 2.17	
						ii	16.87 ± 0.36	
						iii	49.79 ± 0.94	
						iv	32.3 ± 3.4	
						v	36.01 ± 6.6	
							Average	30.04 ± 13.61
Group 3 = 24 - 35 months	Semu	M278S91E61P02	30	Jul-07	30357	i	0	
						ii	0	
						iii	20.37 ± 1.63	

Group 3 = 24 - 35 months	Semu	M278S91E61P02	30	Jul-07	30357	iv	7.41 ± 6.53	
						v	13.99 ± 3.51	
							Average	8.35 ± 8.72
Group 3 = 24 - 35 months	Semu	M278S91E61P02	30	Dec-07	15287	i	0	
						ii	13.37 ± 3.72	
						iii	11.93 ± 0.36	
						iv	11.11 ± 2.47	
						v	14.81 ± 2.83	
							Average	10.25 ± 5.82
Group 3 = 24 - 35 months	Santa Maria	M393S30E41P01	32	Mar-09	12009	i	11.93 ± 0.71	
						ii	22.43 ± 2.49	
						iii	24.07 ± 2.83	
						iv	24.69 ± 0.62	
						v	19.14 ± 2.83	
							Average	20.45 ± 5.17
Group 3 = 24 - 35 months	Sampaka	M450S78E10P01	33	Dec-07	15307	i	42.18 ± 2.49	
						ii	52.06 ± 0.94	
						iii	61.32 ± 1.89	
						iv	71.81 ± 2.92	
						v	78.81 ± 6.77	
							Average	61.23 ± 13.97
Group 3 = 24 - 35 months	Santa Maria	M335S83E2P01	33	Dec-07	15277	i	83.95 ± 2.47	
						ii	78.19 ± 4.02	
						iii	80.04 ± 11.47	
						iv	90.95 ± 7.21	
						v	69.55 ± 5.74	
							Average	80.53 ± 9.32
Group 4 = 36 - 47 months	Bakake Grande	M1292S52E5P01	36	Jul-07	30357	i	43.21 ± 2.83	
						ii	48.77 ± 2.14	
						iii	61.93 ± 5.25	
						iv	44.65 ± 9.1	
						v	72.63 ± 2.78	
							Average	54.24 ± 12.49

Group 4 = 36 - 47 months	Bakake Grande	M1292S63E12P01	36	Nov-08	16498	i	55.97 ± 2.57	
						ii	55.56 ± 4.32	
						iii	66.05 ± 2.47	
						iv	56.17 ± 4.28	
						v	76.95 ± 6.31	
							Average	62.14 ± 9.39
Group 4 = 36 - 47 months	Bakake Grande - Bososo	M1292S51E10P01	36	Jul-07	30357	i	70.99 ± 3.27	
						ii	74.49 ± 4.67	
						iii	71.4 ± 0.94	
						iv	60.91 ± 4.79	
						v	73.46 ± 1.07	
							Average	70.25 ± 5.78
Group 4 = 36 - 47 months	Bososo	M1292S52E5P01	36	Jul-07	30357	i	12.55 ± 1.78	
						ii	29.63 ± 3.44	
						iii	34.57 ± 1.85	
						iv	39.71 ± 6.83	
						v	41.98 ± 3.21	
							Average	31.69 ± 11.33
Group 4 = 36 - 47 months	Sampaka	M450S85E5P01	36	Jan-07	10197	i	29.56 ± 2.03	
						ii	26.62 ± 0.93	
						iii	17.54 ± 0.36	
						iv	26.8 ± 1.33	
						v	31.5 ± 1.33	
							Average	26.4 ± 5.05
Group 4 = 36 - 47 months	Sampaka	M450S85E5P01	36	Jul-07	30357	i	61.73 ± 2.69	
						ii	68.52 ± 2.83	
						iii	78.6 ± 2.17	
						iv	66.46 ± 2.34	
						v	72.43 ± 3.17	
							Average	69.55 ± 6.31
Group 4 = 36 - 47 months	Semu	M278S82E69P01	36	Ilegible	Ilegible	i	0	
						ii	0	
						iii	28.19 ± 1.28	

Group 4 = 36 - 47 months	Semu	[illegible]	36	Ilegible	Ilegible	iv	0	
						v	0	
							Average	5.64 ± 11.68
Group 4 = 36 - 47 months	Semu	M278S91E4P01	36	Ilegible	Ilegible	i	0	
						ii	0	
						iii	0	
						iv	0	
						v	0	
							Average	0
Group 4 = 36 - 47 months	Basopu	M502S39E11P01	37	Nov-06	13646	i	3.2 ± 0.79	
						ii	10.36 ± 0.93	
						iii	31.02 ± 0.94	
						iv	8.86 ± 1.36	
						v	18.04 ± 1.17	
							Average	14.3 ± 9.91
Group 4 = 36 - 47 months	Basopu	M502S39E11P01	37	Jul-07	30357	i	26.64 ± 1.4	
						ii	30.06 ± 1.15	
						iii	52.02 ± 0.87	
						iv	38.16 ± 1.68	
						v	47.14 ± 2.51	
							Average	38.8 ± 10.04
Group 4 = 36 - 47 months	Santa Maria	M394S2E12P01	37	Dec-07	15277	i	9.26 ± 0.62	
						ii	4.94 ± 4.32	
						iii	39.71 ± 2.78	
						iv	17.9 ± 1.63	
						v	26.54 ± 6.5	
							Average	19.67 ± 13.3
Group 4 = 36 - 47 months	Campo Yaunde	M335S27E22P01	38	Dec-07	15307	i	64.4 ± 1.28	
						ii	54.12 ± 3.97	
						iii	60.49 ± 3.44	
						iv	75.93 ± 2.47	
						v	74.69 ± 1.85	
							Average	65.93 ± 8.95

Group 4 = 36 - 47 months	Ela Nguema	M278S30E22P01	38	Jan-07	10197	i	38.48 ± 6.8	
						ii	46.09 ± 2.49	
						iii	53.5 ± 1.55	
						iv	48.56 ± 2.49	
						v	49.59 ± 3.92	
							Average	47.24 ± 6.13
Group 4 = 36 - 47 months	Semu	M336S40E29P01	39	Ilegible	Ilegible	i	42.39 ± 9.77	
						ii	44.24 ± 4.47	
						iii	56.79 ± 5.49	
						iv	55.56 ± 8.07	
						v	52.26 ± 3.04	
							Average	50.25 ± 8.27
Group 4 = 36 - 47 months	Central	M276S69E18P01	40	Jan-07	10197	i	0	
						ii	0	
						iii	0	
						iv	0	
						v	0	
							Average	0
Group 4 = 36 - 47 months	Punta Europa	M211S57E4P01	40	Jul-07	30357	i	4.5 ± 0.56	
						ii	0.86 ± 0.1	
						iii	27.48 ± 1.12	
						iv	5.24 ± 1.37	
						v	21.42 ± 0.92	
							Average	11.9 ± 10.83
Group 4 = 36 - 47 months	Bososo - Bakake Grande	M1292S43E617P1	41	Jul-07	30357	i	0	
						ii	7.41 ± 2.14	
						iii	13.99 ± 3.77	
						iv	7.82 ± 2.49	
						v	12.76 ± 0.36	
							Average	8.4 ± 5.46
Group 4 = 36 - 47 months	Bososo - Bakake Grande	M1292S43E17P1	41	Jul-07	Ilegible	i	14.2 ± 1.63	
						ii	14.2 ± 0.62	
						iii	26.75 ± 1.98	

Group 4 = 36 - 47 months	Bososo - Bakake Grande	[illegible]	41	Jul-07	Ilegible	iv	13.58 ± 2.47	
						v	16.05 ± 1.63	
							Average	16.95 ± 5.36
Group 4 = 36 - 47 months	Campo Yaunde	M335S68E34P01	41	Ilegible	Ilegible	i	0	
						ii	0	
						iii	0	
						iv	0	
						v	0	
							Average	0
Group 4 = 36 - 47 months	Ela Nguema	M279S37E28P01	42	Ilegible	Ilegible	i	64.2 ± 5.35	
						ii	30.45 ± 2.92	
						iii	151.23 ± 14.02	
						iv	154.73 ± 21.91	
						v	298.97 ± 17.04	
							Average	139.92 ± 97.14
Group 4 = 36 - 47 months	Sampaka	M450S85E5P01	42	Jan-07	10197	i	38.68 ± 1.98	
						ii	40.33 ± 3.51	
						iii	57 ± 2.57	
						iv	69.96 ± 1.28	
						v	54.32 ± 5.05	
							Average	52.06 ± 12.25
Group 4 = 36 - 47 months	Santa Maria	M393S30E18P01	42	Jun-06	12280	i	72.72 ± 12.39	
						ii	59.47 ± 9.04	
						iii	96.09 ± 4.99	
						iv	76.13 ± 4.34	
						v	86.42 ± 12.53	
							Average	77.52 ± 14.9
Group 4 = 36 - 47 months	Santa Maria	M335S93E3P01	42	Ilegible	Ilegible	i	0	
						ii	0	
						iii	4.12 ± 3.56	
						iv	0	
						v	9.47 ± 1.28	
							Average	2.72 ± 4.12

Group 4 = 36 - 47 months	Sacriba	M502S37E28P01	43	Nov-06	13646	i	74.28 ± 12.49	
						ii	82.51 ± 2.57	
						iii	93 ± 4.2	
						iv	80.45 ± 2.78	
						v	79.42 ± 5.18	
							Average	81.93 ± 8.45
Group 4 = 36 - 47 months	Sacriba	M502S37E28P01	43	Jul-07	30357	i	94.86 ± 2.57	
						ii	90.95 ± 5.25	
						iii	77.16 ± 3.27	
						iv	96.09 ± 3.04	
						v	88.68 ± 5.93	
							Average	89.55 ± 7.84
Group 4 = 36 - 47 months	Sacriba	M502S37E28P01	43	Dec-07	15307	i	43.21 ± 3.27	
						ii	78.81 ± 4.34	
						iii	89.51 ± 9.32	
						iv	61.52 ± 2.78	
						v	59.88 ± 3.85	
							Average	66.58 ± 17.22
Group 4 = 36 - 47 months	Central	M277S53E20P01	44	Jan-07	10197	i	64.61 ± 2.85	
						ii	77.98 ± 3.62	
						iii	80.45 ± 7.36	
						iv	88.48 ± 5.18	
						v	88.27 ± 7.48	
							Average	79.96 ± 10.21
Group 4 = 36 - 47 months	Santa Maria	M394S12E77P01	45	Ilegible	Ilegible	i	28.6 ± 4.2	
						ii	21.6 ± 3.44	
						iii	32.72 ± 5.27	
						iv	26.13 ± 0.94	
						v	28.19 ± 2.17	
							Average	27.45 ± 4.8
Group 5 = 48 - 52 months	Punta Europa	M211S57E4P01	48	Jan-07	10197	i	2.8 ± 0.39	
						ii	8.4 ± 1.66	
						iii	10.38 ± 0.69	

Group 5 = 48 - 52 months	Punta Europa	M211S57E4P01	48	Jan-07	10197	iv	7.98 ± 1.35
						v	29.9 ± 1.23
						Average	11.89 ± 9.64
Group 5 = 48 - 52 months	Punta Europa	M211S5E4P01	48	Jul-07	30357	i	0.44 ± 0.1
						ii	0.36 ± 0.1
						iii	1 ± 1.04
						iv	1.78 ± 0.92
						v	6.32 ± 0.81
						Average	1.98 ± 2.37
Group 5 = 48 - 52 months	Sampaka	M509S27E59P01	48	Jan-07	10197	i	71.19 ± 3.04
						ii	65.43 ± 4.45
						iii	48.77 ± 8.75
						iv	63.17 ± 5.03
						v	73.05 ± 0.71
						Average	64.32 ± 9.88
Group 5 = 48 - 52 months	Sampaka	M509S26E46P01	48	Jan-07	10197	i	28.19 ± 1.78
						ii	32.1 ± 4.32
						iii	59.26 ± 1.23
						iv	61.52 ± 4.79
						v	59.05 ± 5.74
						Average	48.02 ± 15.56
Group 5 = 48 - 52 months	Semu	M366S17E132P01	48	Dec-07	15277	i	59.26 ± 3.31
						ii	38.58 ± 8.37
						iii	59.96 ± 4.82
						iv	58.5 ± 3.28
						v	55.62 ± 2.38
						Average	54.38 ± 9.33
Group 5 = 48 - 52 months	Semu	M277S71E88P02	48	Nov-08	Ilegible	i	37.86 ± 1.28
						ii	35.8 ± 1.85
						iii	35.19 ± 4.05
						iv	16.17 ± 6.44
						v	31.07 ± 3.97
						Average	29.45 ± 9.88

Group 5 = 48 - 52 months	Semu - Alcaide	M337S95E62P01	48	Jul-07	30357	i	4.94 ± 0.87	
						ii	3.86 ± 0.78	
						iii	55.09 ± 4.43	
						iv	9.1 ± 0.78	
						v	42.59 ± 13.96	
							Average	23.12 ± 22.77
Group 5 = 48 - 52 months	Semu - Alcaide	M336S17E132P01	48	Jul-07	30357	i	36.63 ± 2.85	
						ii	3.5 ± 1.89	
						iii	34.57 ± 1.63	
						iv	34.16 ± 3.51	
						v	26.17 ± 10.66	
							Average	26.91 ± 13.16
Group 5 = 48 - 52 months	Semu - Alcaide	M336S17E132P01	48	Jul-07	30357	i	20.99 ± 4.66	
						ii	16.79 ± 12.94	
						iii	51.23 ± 4.32	
						iv	44.44 ± 12.33	
						v	43.95 ± 11.63	
							Average	34.41 ± 17.15
Group 5 = 48 - 52 months	Semu - Alcaide	M336S40E30P01	48	Dec-07	15287	i	4.12 ± 0.36	
						ii	3.29 ± 0.36	
						iii	58.23 ± 3.56	
						iv	14.81 ± 3.09	
						v	34.77 ± 4.34	
							Average	23.05 ± 21.81
Group 5 = 48 - 52 months	Semu - Alcaide	M337S95E62P01	48	Ilegible	Ilegible	i	75.51 ± 6.77	
						ii	67.08 ± 5.87	
						iii	69.75 ± 7.12	
						iv	61.11 ± 3.44	
						v	72.02 ± 3.17	
							Average	69.09 ± 6.86
Group 5 = 48 - 52 months	Semu - Alcaide	M336S10E23P01	48	Dec-07	15297	i	6.79 ± 0.62	
						ii	0	
						iii	19.14 ± 1.07	

Group 5 = 48 - 52 months	Semu - Alcaide	M336S10E23P01	48	Dec-07	15297	iv	6.58 ± 1.43	
						v	20.99 ± 4.45	
							Average	10.7 ± 8.53
Group 5 = 48 - 52 months	Semu - Alcaide	M336S29E5P01	48	Dec-07	15277	i	43.64 ± 3.06	
						ii	41.86 ± 1.52	
						iii	66.46 ± 2.15	
						iv	41.4 ± 2.38	
						v	48.9 ± 1.38	
							Average	48.45 ± 9.83
Group 5 = 48 - 52 months	Basopu	M502S556E13P01	50	Jul-07	30357	i	21.84 ± 1.22	
						ii	27.98 ± 1.83	
						iii	32.64 ± 2.33	
						iv	33.16 ± 0.67	
						v	38.94 ± 3.94	
							Average	30.91 ± 6.21
Group 5 = 48 - 52 months	Basopu	M502S56E13P01	50	Jan-07	10197	i	19.6 ± 3.43	
						ii	20.83 ± 4.92	
						iii	9.41 ± 1.05	
						iv	17.9 ± 2.62	
						v	15.74 ± 1.07	
							Average	16.7 ± 4.92
Group 5 = 48 - 52 months	Campo Yaunde	M335S37E77P01	50	Jan-07	10197	i	7.28 ± 0.24	
						ii	13.26 ± 1.65	
						iii	7.6 ± 1.62	
						iv	18.92 ± 2.29	
						v	7.82 ± 1.27	
							Average	10.98 ± 4.87
Group 5 = 48 - 52 months	Campo Yaunde	M335S37E77P01	50	Ilegible	10197	i	2.28 ± 0.59	
						ii	3.54 ± 3.48	
						iii	0.36 ± 0.1	
						iv	0.48 ± 0	
						v	0.3 ± 0.18	
							Average	1.59 ± 2.23

Group 5 = 48 - 52 months	Campo Yaunde	M335S37E77P01	50	no tiene	no tiene	i	3.76 ± 0.31	
						ii	1.48 ± 0.91	
						iii	5.52 ± 0.67	
						iv	5.56 ± 0.35	
						v	6.48 ± 0.7	
							Average	4.56 ± 1.9
Group 5 = 48 - 52 months	Campo Yaunde	M335S30E24P02	50	Jan-07	10197	i	61.93 ± 2.92	
						ii	55.76 ± 4.47	
						iii	84.57 ± 10.82	
						iv	65.43 ± 11.43	
						v	84.16 ± 7.77	
							Average	70.37 ± 14.08
Group 5 = 48 - 52 months	Campo Yaunde	M335S30E65P01	50	Jul-07	30357	i	51.65 ± 5.39	
						ii	38.48 ± 3.04	
						iii	66.26 ± 6.77	
						iv	39.3 ± 6.18	
						v	62.55 ± 5.6	
							Average	51.65 ± 12.77
Group 5 = 48 - 52 months	Central	M276S69E103P01	50	Jan-07	10197	i	1.04 ± 0.15	
						ii	1.32 ± 0.6	
						iii	0.7 ± 0.24	
						iv	0.56 ± 0.07	
						v	0.46 ± 0.19	
							Average	0.82 ± 0.43
Group 5 = 48 - 52 months	Rebola	M459S89E2P01	50	Jul-07	30357	i	60.78 ± 2.56	
						ii	65.78 ± 2.9	
						iii	82.94 ± 3.72	
						iv	72.58 ± 1.76	
						v	80.1 ± 2.18	
							Average	72.44 ± 8.91
Group 5 = 48 - 52 months	Santa Maria	M333S95E42P01	50	Ilegible	Ilegible	i	3.09 ± 1.23	
						ii	4.12 ± 1.28	
						iii	3.09 ± 0.62	

Group 5 = 48 - 52 months	Santa Maria	[illegible]	50	Ilegible	Ilegible	iv	4.32 ± 1.63	
						v	3.7 ± 0.62	
							Average	3.66 ± 1.11
Group 5 = 48 - 52 months	Santa Maria	M335S92E15P01	50	Jul-07	30357	i	22.22 ± 1.23	
						ii	28.19 ± 0.94	
						iii	41.56 ± 3.97	
						iv	12.96 ± 3.49	
						v	20.99 ± 0.62	
							Average	23.75 ± 10.33
Group 5 = 48 - 52 months	Central	M276S89E114P01	51	Jan-07	10197	i	38.68 ± 0.94	
						ii	31.48 ± 1.23	
						iii	38.27 ± 6.96	
						iv	41.98 ± 6.96	
						v	50.82 ± 3.17	
							Average	40.25 ± 7.62
Group 5 = 48 - 52 months	Central	M276S68E34P01	51	Dec-07	15297	i	26.54 ± 1.63	
						ii	27.57 ± 4.95	
						iii	37.65 ± 1.07	
						iv	24.9 ± 5.74	
						v	51.85 ± 5.49	
							Average	33.7 ± 11.07
Group 5 = 48 - 52 months	Ela Nguema	M278S8E61P01	51	Jul-07	30357	i	0	
						ii	21.4 ± 2.49	
						iii	41.98 ± 4.32	
						iv	25.1 ± 3.97	
						v	36.21 ± 4.71	
							Average	22.43 ± 18.52

cording to the age of the LLINs

Test Kit category	Mortality rate (%)	
Status & DM range (mg/m^2)	Each section	Whole net
Pass (>25)		
Pass (>25)		
Pass (>25)		
Pass (>25)		
Pass (>25)		
Pass (>25)		
Pass (>25)		
Pass (>25)		
Pass (>25)		
Pass (>25)		
Pass (>25)		
Pass (>25)		
Pass (>25)		
Pass (>25)		
Pass (>25)		
Pass (>25)		
Pass (>25)	100	
Pass (>25)	100	
Pass (>25)	100	
Pass (>25)	90	
		97.5
Fail (5-15)		
Fail (5-15)	60	
Pass (>25)	90	

Fail (5-15)	70	
Pass (15-25)	80	
		75
Pass (>25)		
Pass (>25)		
Pass (>25)		
Pass (>25)		
Pass (>25)		
Pass (>25)		
Pass (15-25)	100	
Pass (>25)	100	
Pass (15-25)	90	
Pass (>25)	90	
		95
Pass (>25)		
Fail (5-15)		
Pass (>25)		
Pass (>25)		
Pass (>25)		
Pass (>25)		
Pass (>25)	100	
Pass (>25)	90	
Pass (>25)	90	
Pass (>25)	90	
		92.5
Pass (>25)		
Pass (>25)	100	
Pass (>25)	100	
Pass (>25)	100	
Pass (>25)	100	
		100

Pass (>25)		
Pass (>25)		
Pass (>25)		
Pass (>25)		
Pass (>25)		
Pass (>25)		
Pass (>25)	100	
Pass (>25)	100	
Pass (>25)	90	
Pass (>25)	90	
		95
Pass (>25)		
Pass (>25)		
Pass (>25)		
Pass (>25)		
Pass (>25)		
Pass (>25)		
Pass (>25)	100	
Pass (>25)	100	
Pass (>25)	90	
Pass (>25)	100	
		97.5
Pass (>25)		
Pass (15-25)	100	
Pass (>25)	100	
Pass (>25)	100	
Pass (>25)	100	
		100
Fail (0-5)		
Pass (15-25)		
Pass (>25)		

Pass (>25)		
Pass (>25)		
Pass (>25)		
Pass (>25)		
Pass (>25)		
Pass (>25)		
Pass (>25)		
Pass (>25)		
Pass (>25)		
Pass (>25)		
Pass (>25)		
Pass (>25)		
Pass (>25)		
Pass (>25)		
Pass (>25)		
Pass (>25)		
Pass (>25)		
Pass (>25)		
Pass (>25)	100	
Pass (>25)	100	
Pass (>25)	100	
Pass (>25)	100	
		100
Pass (15-25)		
Fail (5-15)	100	
Fail (5-15)	100	
Pass (>25)	100	
Pass (>25)	100	
		100

Fail (5-15)		
Pass (15-25)	100	
Pass (>25)	100	
Pass (>25)	90	
Pass (>25)	100	
		97.5
Pass (>25)		
Pass (>25)		
Pass (>25)		
Pass (>25)		
Pass (>25)		
Pass (>25)		
Pass (15-25)	100	
Pass (>25)	100	
Pass (>25)	100	
Pass (>25)	100	
		100
Pass (>25)		
Pass (>25)	100	
Pass (>25)	100	
Pass (>25)	100	
Pass (>25)	100	
		100
Pass (>25)		
Pass (>25)	100	
Pass (>25)	100	
Pass (>25)	100	
Pass (>25)	100	
		100
Pass (>25)		
Pass (>25)		
Pass (>25)		

Pass (>25)		
Pass (>25)		
Pass (15-25)		
Pass (>25)		
Pass (>25)		
Pass (>25)		
Pass (>25)		
Pass (>25)		
Pass (>25)	100	
Pass (>25)	100	
Pass (>25)	90	
Pass (>25)	80	
		92.5
Pass (>25)		
Pass (>25)	100	
Pass (>25)	80	
Pass (>25)	90	
Pass (>25)	100	
		92.5
Pass (>25)		
Pass (>25)		
Pass (>25)		
Pass (>25)		
Pass (>25)		
Fail (0)		
Fail (5-15)	70	
Pass (>25)	50	
Pass (15-25)	80	
Pass (>25)	50	
		62.5

Fail (0)		
Fail (0)	70	
Pass (>25)	100	
Fail (5-15)	60	
Fail (5-15)	80	
		77.5
Fail (0)		
Pass (>25)		
Pass (>25)		
Pass (>25)		
Pass (>25)		
Pass (>25)		
Pass (>25)	100	
Pass (>25)	90	
Pass (>25)	100	
Pass (>25)	90	
		95
Pass (15-25)		
Pass (>25)		
Pass (>25)		
Pass (>25)		
Pass (>25)		
Fail (5-15)		
Fail (0)	70	
Pass (>25)	60	
Fail (0)	100	
Pass (15-25)	70	
		75
Pass (>25)		
Pass (>25)	100	
Pass (>25)	100	

Pass (>25)	100	
Pass (>25)	100	
		100
Fail (5-15)		
Pass (>25)	90	
Pass (>25)	90	
Pass (15-25)	90	
Pass (>25)	100	
		92.5
Pass (15-25)		
Fail (5-15)		
Pass (15-25)		
Pass (>25)		
Fail (5-15)		
Fail (5-15)		
Fail (5-15)		
Pass (15-25)		
Fail (5-15)		
Pass (>25)		
Pass (>25)		
Pass (>25)		
Pass (>25)		
Pass (>25)		
Pass (>25)		
Pass (>25)		
Pass (>25)		
Pass (>25)		
Pass (>25)		
Pass (>25)		

Pass (>25)		
Pass (>25)		
Pass (>25)		
Pass (>25)		
Pass (>25)		
Pass (>25)		
Pass (>25)	80	
Pass (>25)	80	
Pass (>25)	100	
Pass (>25)	100	
		90
Fail (5-15)		
Pass (15-25)		
Pass (15-25)		
Fail (5-15)		
Pass (15-25)		
Pass (>25)		
Pass (>25)		
Pass (>25)		
Pass (>25)		
Pass (>25)		
Pass (>25)		
Pass (>25)		
Pass (>25)		
Pass (>25)		
Pass (>25)		
Pass (>25)		
Pass (>25)		
Pass (>25)		

Pass (>25)		
Pass (>25)		
Pass (>25)		
Pass (>25)		
Pass (>25)		
Pass (>25)		
Pass (>25)		
Pass (>25)		
Pass (>25)		
Pass (>25)		
Pass (>25)		
Pass (>25)		
Pass (>25)		
Pass (>25)	90	
Pass (>25)	90	
Pass (>25)	100	
Pass (>25)	90	
		92.5
Pass (>25)		
Pass (>25)		
Pass (>25)		
Pass (>25)		
Pass (>25)		
Pass (>25)		
Pass (>25)	100	
Pass (>25)	80	
Pass (>25)	100	
Pass (>25)	90	
		92.5

Pass (>25)		
Fail (5-15)		
Pass (>25)		
Pass (>25)		
Pass (>25)		
Pass (15-25)		
Pass (>25)		
Pass (>25)		
Pass (>25)		
Pass (>25)		
Pass (>25)		
Pass (>25)	80	
Pass (>25)	100	
Pass (>25)	90	
Pass (>25)	90	
		90
Pass (>25)		
Pass (>25)	90	
Pass (>25)	90	
Pass (>25)	90	
Pass (>25)	80	
		87.5
Fail (5-15)		
Pass (15-25)		
Pass (>25)		
Pass (>25)		
Pass (>25)		
Pass (>25)		
Pass (>25)		
Fail (5-15)		

Pass (>25)		
Pass (>25)		
Pass (>25)		
Pass (>25)		
Pass (>25)		
Pass (>25)		
Pass (>25)		
Pass (>25)		
Pass (>25)		
Pass (>25)		
Pass (>25)		
Pass (>25)		
Fail (0)		
Fail (0)		
Pass (15-25)		
Pass (>25)		
Pass (>25)		
Fail (5-15)		
Fail (0)		
Pass (15-25)		
Fail (5-15)		
Pass (15-25)		
Pass (15-25)		
Fail (0)		
Pass (15-25)		
Pass (15-25)		
Pass (>25)		

Pass (15-25)		
Pass (>25)		
Pass (>25)		
Pass (>25)		
Pass (>25)		
Pass (15-25)		
Pass (15-25)	90	
Pass (15-25)	100	
Pass (>25)	90	
Pass (>25)	80	
		90
Fail (0)		
Fail (0)		
Fail (0)		
Fail (0)		
Fail (0)		
Pass (15-25)		
Pass (15-25)		
Pass (15-25)		
Pass (15-25)		
Fail (5-15)		
Pass (15-25)		
Pass (15-25)	80	
Pass (>25)	80	
Pass (>25)	90	
Pass (>25)	90	
		85
Fail (0)		
Fail (0)	60	
Pass (15-25)	80	

Fail (5-15)	40	
Fail (5-15)	70	
		62.5
Fail (0)		
Fail (5-15)		
Fail (5-15)		
Fail (5-15)		
Fail (5-15)		
Fail (5-15)		
Pass (15-25)	90	
Pass (15-25)	70	
Pass (15-25)	80	
Pass (15-25)	80	
		80
Pass (>25)		
Pass (>25)		
Pass (>25)		
Pass (>25)		
Pass (>25)		
Pass (>25)		
Pass (>25)	80	
Pass (>25)	100	
Pass (>25)	100	
Pass (>25)	100	
		95
Pass (>25)		
Pass (>25)		
Pass (>25)		
Pass (>25)		
Pass (>25)		

Pass (>25)		
Pass (>25)		
Pass (>25)		
Pass (>25)		
Pass (>25)		
Pass (>25)		
Pass (>25)		
Pass (>25)		
Pass (>25)		
Pass (>25)		
Fail (5-15)		
Pass (>25)	80	
Pass (>25)	100	
Pass (>25)	90	
Pass (>25)	100	
		92.5
Pass (>25)		
Pass (>25)	90	
Pass (15-25)	70	
Pass (>25)	80	
Pass (>25)	100	
		85
Pass (>25)		
Pass (>25)	90	
Pass (>25)	100	
Pass (>25)	100	
Pass (>25)	100	
		97.5
Fail (0)		
Fail (0)		
Pass (>25)		

Fail (0)<br>Fail (0)		
Fail (0)<br>Fail (0)<br>Fail (0)<br>Fail (0)<br>Fail (0)		
Fail (0-5)<br>Fail (5-15)<br>Pass (>25)<br>Fail (5-15)<br>Pass (15-25)	 50<br>30<br>30<br>40	
		37.5
Pass (>25)<br>Pass (>25)<br>Pass (>25)<br>Pass (>25)<br>Pass (>25)	 100<br>80<br>70<br>100	
		87.5
Fail (5-15)<br>Fail (0-5)<br>Pass (>25)<br>Pass (15-25)<br>Pass (>25)		
Pass (>25)<br>Pass (>25)<br>Pass (>25)<br>Pass (>25)<br>Pass (>25)		

Pass (>25)		
Pass (>25)	90	
Pass (>25)	90	
Pass (>25)	100	
Pass (>25)	100	
		95
Pass (>25)		
Pass (>25)		
Pass (>25)		
Pass (>25)		
Pass (>25)		
Fail (0)		
Fail (0)		
Fail (0)		
Fail (0)		
Fail (0)		
Fail (0-5)		
Fail (0-5)	40	
Pass (>25)	40	
Fail (5-15)	20	
Pass (15-25)	20	
		30
Fail (0)		
Fail (5-15)	50	
Fail (5-15)	70	
Fail (5-15)	40	
Fail (5-15)	70	
		57.5
Fail (5-15)		
Fail (5-15)	60	
Pass (>25)	70	

Fail (5-15)	60	
Pass (15-25)	80	
		67.5
Fail (0)		
Fail (0)		
Fail (0)		
Fail (0)		
Fail (0)		
Pass (>25)		
Pass (>25)		
Pass (>25)		
Pass (>25)		
Pass (>25)		
Pass (>25)		
Pass (>25)		
Pass (>25)		
Pass (>25)		
Pass (>25)		
Pass (>25)		
Pass (>25)		
Pass (>25)		
Pass (>25)		
Pass (>25)		
Fail (0)		
Fail (0)		
Fail (0-5)		
Fail (0)		
Fail (5-15)		

Pass (>25)		
Pass (>25)		
Pass (>25)		
Pass (>25)		
Pass (>25)		
Pass (>25)		
Pass (>25)		
Pass (>25)		
Pass (>25)		
Pass (>25)		
Pass (>25)		
Pass (>25)		
Pass (>25)		
Pass (>25)		
Pass (>25)		
Pass (>25)		
Pass (>25)		
Pass (>25)		
Pass (>25)		
Pass (>25)		
Pass (>25)		
Pass (15-25)		
Pass (>25)		
Pass (>25)		
Pass (>25)		
Fail (0-5)		
Fail (5-15)	60	
Fail (5-15)	50	

Fail (5-15)	40	
Pass (>25)	60	
		52.5
Fail (0-5)		
Fail (0-5)		
Fail (0-5)		
Fail (0-5)		
Fail (5-15)		
Pass (>25)		
Pass (>25)		
Pass (>25)		
Pass (>25)		
Pass (>25)		
Pass (>25)		
Pass (>25)		
Pass (>25)		
Pass (>25)		
Pass (>25)		
Pass (>25)		
Pass (>25)	100	
Pass (>25)	90	
Pass (>25)	100	
Pass (>25)	100	
		97.5
Pass (>25)		
Pass (>25)		
Pass (>25)		
Pass (15-25)		
Pass (>25)		

Fail (0-5)		
Fail (0-5)		
Pass (>25)		
Fail (5-15)		
Pass (>25)		
Pass (>25)		
Fail (0-5)		
Pass (>25)		
Pass (>25)		
Pass (>25)		
Pass (15-25)		
Pass (15-25)	90	
Pass (>25)	100	
Pass (>25)	100	
Pass (>25)	100	
		97.5
Fail (0-5)		
Fail (0-5)		
Pass (>25)		
Fail (5-15)		
Pass (>25)		
Pass (>25)		
Pass (>25)		
Pass (>25)		
Pass (>25)		
Pass (>25)		
Fail (5-15)		
Fail (0)		
Pass (15-25)		

Fail (5-15)		
Pass (15-25)		
Pass (>25)		
Pass (>25)	100	
Pass (>25)	80	
Pass (>25)	90	
Pass (>25)	100	
		92.5
Pass (15-25)		
Pass (>25)	70	
Pass (>25)	100	
Pass (>25)	90	
Pass (>25)	100	
		90
Pass (15-25)		
Pass (15-25)		
Fail (5-15)		
Pass (15-25)		
Pass (15-25)		
Fail (5-15)		
Fail (5-15)	20	
Fail (5-15)	70	
Pass (15-25)	10	
Fail (5-15)	30	
		32.5
Fail (0-5)		
Fail (0-5)		
Fail (0-5)		
Fail (0-5)		
Fail (0-5)		

Fail (0-5)		
Fail (0-5)		
Fail (5-15)		
Fail (5-15)		
Fail (5-15)		
Pass (>25)		
Pass (>25)		
Pass (>25)		
Pass (>25)		
Pass (>25)		
Pass (>25)		
Pass (>25)	100	
Pass (>25)	90	
Pass (>25)	100	
Pass (>25)	100	
		97.5
Fail (0-5)		
Fail (0-5)		
Fail (0-5)		
Fail (0-5)		
Fail (0-5)		
Pass (>25)		
Pass (>25)	90	
Pass (>25)	100	
Pass (>25)	100	
Pass (>25)	100	
		97.5
Fail (0-5)		
Fail (0-5)		
Fail (0-5)		

Fail (0-5)<br>Fail (0-5)		
Pass (15-25)<br>Pass (>25)<br>Pass (>25)<br>Fail (5-15)<br>Pass (15-25)		
Pass (>25)<br>Pass (>25)<br>Pass (>25)<br>Pass (>25)<br>Pass (>25)		
Pass (>25)<br>Pass (>25)<br>Pass (>25)<br>Pass (15-25)<br>Pass (>25)		
Fail (0)<br>Pass (15-25)<br>Pass (>25)<br>Pass (>25)<br>Pass (>25)	 80<br>80<br>50<br>100	
		77.5
